# Dynamic energy conversion in protein catalysis: From brownian motion to enzymatic function

**DOI:** 10.1016/j.csbj.2025.07.050

**Published:** 2025-07-30

**Authors:** Sarfaraz K. Niazi

**Affiliations:** University of Illinois, Chicago, IL 60612, USA

**Keywords:** Protein structure, Dynamic modeling, Enzymatic activity, Quantum limitations, Drug discovery

## Abstract

Recent advances in computational biology and experimental techniques reveal that enzymatic catalysis fundamentally depends on proteins' ability to harness thermal energy through conformational fluctuations. Rather than functioning as rigid molecular locks, proteins operate as dynamic machines that continuously sample different structural states, with α-helices and β-sheets acting as sophisticated energy transduction elements that capture Brownian motion and channel it toward productive chemical transformations. Molecular dynamics simulations, combined with machine learning tools such as AlphaFold, demonstrate that these conformational dynamics directly modulate substrate binding affinity and reaction pathway selection, suggesting that proteins actively convert environmental thermal noise into catalytic work rather than merely stabilizing transition states. This dynamic energy conversion paradigm fundamentally reshapes our approach to pharmaceutical design and enzyme engineering by emphasizing the targeting of conformational ensembles rather than static structures, while also raising important questions about the universal applicability of this mechanism across all enzyme classes and the experimental methodologies needed to validate dynamic catalytic models. The shift from viewing proteins as passive structural scaffolds to active energy converters represents a transformative reconceptualization of biological catalysis with far-reaching implications for our understanding of life's molecular machinery.

## Graphical Abstract

**Figure:**
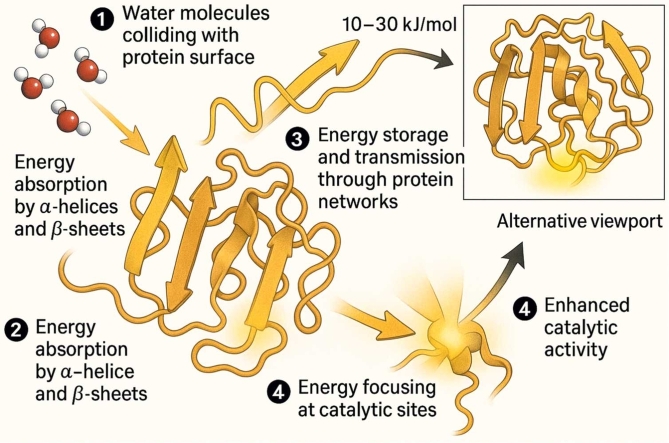

## 1 Introduction and historical context

### 1.1 The crisis in static structural biology

The classical doctrine linking protein structure to function through static three-dimensional conformations has profoundly shaped the trajectory of biochemistry for generations, yielding transformative methodologies such as X-ray crystallography, cryo-electron microscopy, and NMR spectroscopy that have populated the Protein Data Bank with more than 200,000 molecular architectures [1]. Yet this crystallographic worldview inherently constrains our comprehension of enzymatic behavior by freezing proteins into thermodynamically favorable poses under artificial conditions—cryogenic temperatures, dehydrated environments, and crystal lattice restrictions—that bear little resemblance to physiological reality, producing isolated snapshots that obscure the fluid choreography of molecular motion essential to catalysis.

### 1.2 Evolution of dynamic perspectives

The paradigm's insufficiency emerges starkly when confronting the observation by Henzler-Wildman and Kern [2] that proteins constitute "dynamic ensembles" perpetually exploring conformational landscapes rather than occupying fixed coordinates, with these structural fluctuations and ephemeral transition states proving indispensable for understanding how enzymes achieve their remarkable catalytic prowess and substrate selectivity in living systems.

The gradual awakening to protein dynamics as a central force in enzymatic catalysis has unfolded across multiple scientific generations, beginning with theoretical frameworks proposing that conformational flexibility enables the sequential choreography of substrate capture, chemical transformation, and product expulsion, culminating in Koshland's revolutionary "induced fit" hypothesis [3] which fundamentally disrupted Fischer's 1894 rigid "lock and key" doctrine by demonstrating that proteins actively reshape themselves in response to substrate encounters rather than maintaining static complementarity.

Experimental evidence supporting the importance of protein dynamics began accumulating in the 1970s. Relaxation studies, hydrogen-deuterium exchange experiments, and fluorescence spectroscopy revealed that proteins exhibit motion at multiple timescales, from rapid bond vibrations to slower domain movements. The pioneering work of Frauenfelder and colleagues [4] demonstrated that proteins do not occupy single conformational states but rather sample ensembles of conformations driven by thermal energy.

### 1.3 The revolutionary hypothesis: proteins as brownian energy harvesters

A mechanistic framework emerging from these foundational insights reconceptualizes enzymatic function through the lens of active energy conversion, where proteins—as proposed by Cao and Ding and substantiated by accumulating experimental validation [5]—operate as molecular transducers that capture and redirect the chaotic kinetic energy of Brownian motion into productive chemical transformations, fundamentally reframing catalysis as a dynamic energy harvesting process rather than mere structural complementarity.

### 1.4 Core principles and scientific debate

#### 1.4.1 Core principles of dynamic energy conversion

The fundamental principles underlying dynamic energy conversion in proteins encompass a sophisticated cascade of molecular events that transform random thermal fluctuations into directed catalytic work. This conceptual framework requires understanding how proteins absorb energy from their aqueous environment, store this energy through structural deformations, and ultimately utilize it to reduce activation barriers for chemical reactions. The process begins at the femtosecond timescale with water molecules colliding with protein surfaces at frequencies exceeding 10 ¹ ¹ impacts per second, delivering discrete packets of kinetic energy that induce localized conformational perturbations. These initial disturbances propagate through the protein architecture via multiple mechanisms, including hydrogen bond networks, rearrangements of the hydrophobic core, and collective vibrational modes. The absorbed energy is temporarily stored in various structural elements, with α-helices functioning as molecular springs that can store 10–30 kJ/mol through axial compression and bending deformations. At the same time, β-sheets act as planar energy platforms that accommodate shear and stretching forces. This stored mechanical energy subsequently becomes available for catalytic assistance through several mechanisms: direct mechanical strain on substrate bonds, electrostatic field modulation at active sites, dynamic hydrogen bonding networks that stabilize transition states, and conformational changes that optimize catalytic geometry. The entire process represents a sophisticated energy management system where proteins harvest environmental thermal energy and focus it spatially and temporally to achieve remarkable catalytic rate enhancements. Understanding these principles is essential for appreciating how evolution has transformed simple polypeptide chains into sophisticated molecular machines capable of accelerating chemical reactions by factors of 10 ¹ ⁷ or more.

This theoretical model fundamentally reconceptualizes proteins not as passive scaffolds for positioning reactive groups, but as active mechanical systems that directly contribute energy to catalytic reactions.

#### 1.4.2 Scientific perspective box: alternative viewpoints on protein motion in catalysis

The scientific community's perspective on the role of protein dynamics in catalysis reflects the inherent complexity of enzymatic mechanisms and the challenges in experimentally dissecting the contributions of conformational motion to catalytic efficiency. The traditional view, still held by some researchers, maintains that protein dynamics are merely correlated with catalysis rather than causally linked, arguing that the primary role of protein structure is to create an optimal static arrangement of catalytic residues and that observed motions are incidental byproducts of thermal fluctuations. This perspective is supported by the success of static structure-based drug design and the ability to predict enzymatic function solely from crystallographic structures. A moderate dynamic view, gaining increasing acceptance, acknowledges that conformational flexibility enables function by allowing proteins to sample different states required for substrate binding, catalysis, and product release. However, proponents remain uncertain about whether dynamics contribute energy directly to the reaction or simply facilitate access to catalytically competent conformations. The dynamic energy conversion model presented here represents the most radical reconceptualization, proposing that proteins actively harvest and channel thermal energy to power catalytic reactions, with quantitative correlations between dynamics and activity supporting this mechanism. An integrative perspective, perhaps most reflective of biological reality, suggests that multiple mechanisms operate simultaneously, with the relative importance of static positioning versus dynamic contributions varying among enzyme families and reaction types. These diverse viewpoints highlight the ongoing scientific debate and underscore the need for continued experimental and theoretical investigations to elucidate the mechanistic basis of enzymatic catalysis fully. The resolution of this debate has profound implications for drug design, enzyme engineering, and our fundamental understanding of how life achieves its remarkable chemical transformations.

### 1.5 Objectives and methodological framework

This critical examination dissects the revolutionary conceptualization of proteins as dynamic energy transducers in catalysis through comprehensive analysis of theoretical underpinnings, experimental and computational validation, and transformative implications for biotechnology and medicine, employing a systematic methodology that integrates theoretical framework development with empirical verification, computational simulation, and practical implementation across diverse temporal scales as delineated in Table 1, Table 2, Table 3, ultimately synthesizing how this paradigm shift redefines our fundamental understanding of enzymatic function from passive structural templates to active Brownian energy harvesters [20], [21], [22], [23].

**Table 1:** Fundamental principles of dynamic energy conversion in proteins.

**Principle**	**Process**	**Energy Source**	**Energy Range (kJ/mol)**	**Timescale**	**Reference**
Energy Absorption	Water-protein collisions	Brownian motion	0.1–5 per collision	10⁻¹ ⁵−10⁻¹ ² s	[6], [7]
Energy Storage	Structural deformation	Potential energy in bonds	10–30 total	10⁻¹ ²−10⁻⁹ s	[8], [9]
Energy Utilization	Catalytic assistance	Mechanical work	20–40 barrier reduction	10⁻⁹−10⁻⁶ s	[10], [11]

**Table 2:** Scientific perspectives on protein dynamics in catalysis.

**Viewpoint**	**Core Principle**	**Evidence Type**	**Limitations**	**Representative References**
Traditional View	Dynamics are correlated, not causal	Static structure-function relationships	Limited mechanistic insight	[12], [13]
Moderate Dynamic View	Flexibility enables function	Conformational selection studies	Unclear energy contribution	[14], [15]
Dynamic Energy Conversion	Motion directly contributes energy	Quantitative dynamics-activity correlations	Technical measurement challenges	[5], [16], [17]
Integrative Model	Multiple mechanisms operate	Multi-technique validation	Complexity of interpretation	[18], [19]

**Table 3:** Review objectives and methodological approaches.

**Objective**	**Approach**	**Expected Outcome**	**Timeline**	**References**
Theoretical framework development	Mathematical modeling, thermodynamic analysis	Quantitative energy conversion models	2–3 years	[20], [21]
Experimental validation	Multi-technique integration	Correlation coefficients > 0.7	3–5 years	[22], [23]
Computational predictions	MD simulations, AI integration	Structure-dynamics relationships	1–2 years	[24], [25]
Biotechnology applications	Enzyme engineering, drug design	Enhanced catalytic efficiency	5–10 years	[26], [27]

The methodological approach adopted in this review encompasses four interconnected objectives that progressively build understanding from theoretical foundations to practical applications. The aim of developing the theoretical framework is to establish quantitative models that describe how proteins convert thermal energy into catalytic work, requiring the integration of statistical mechanics, molecular dynamics theory, and enzyme kinetics to create predictive equations linking conformational fluctuations to reaction rates [20], [21]. This theoretical work provides the mathematical foundation necessary for understanding energy flow through protein structures and establishes testable hypotheses about the relationship between structural dynamics and catalytic efficiency. The experimental validation objective aims to confirm theoretical predictions through multi-technique approaches, including NMR relaxation dispersion to measure conformational exchange rates, single-molecule fluorescence to observe individual protein dynamics, and temperature-jump experiments to capture transient intermediates [22], [23]. These diverse experimental approaches are essential for capturing dynamics across the full range of relevant timescales from picoseconds to milliseconds. The computational predictions objective leverages molecular dynamics simulations and emerging AI tools to model energy propagation through protein structures and predict how mutations affect dynamic properties [24], [25]. This computational work bridges the gap between atomic-level structural information and macroscopic catalytic behavior. The objective of biotechnology applications is to translate fundamental understanding into practical outcomes through rational enzyme engineering for enhanced industrial catalysts and dynamic drug design, targeting conformational ensembles rather than static structures [26], [27]. Each objective builds upon the previous ones, creating a comprehensive framework for understanding and exploiting dynamic energy conversion in proteins. The timeline and resource requirements for each objective reflect the technical challenges and interdisciplinary nature of this research program.

## 2 Theoretical foundation of energy conversion

### 2.1 The physics of brownian bombardment

The phenomenon of Brownian motion—characterized by Robert Brown in 1827 and mechanistically elucidated by Albert Einstein in 1905 as stochastic particle trajectories driven by molecular collisions—has undergone radical reinterpretation from biological noise to fundamental energy resource, with accumulating evidence revealing that evolution has sculpted proteins to exploit rather than resist these thermal fluctuations [28], as proteins immersed in aqueous environments endure relentless molecular bombardment at rates exceeding thousands of water molecule impacts per microsecond according to Di Rienzo et al. [28], where each microscopic collision transfers momentum that induces localized structural perturbations which, though individually negligible, collectively orchestrate substantial conformational waves that ripple through entire protein architectures, transforming random thermal agitation into directed functional motion.

**The quantitative framework for this process can be expressed as:**

The collision frequency (ν) between water molecules and protein surfaces follows:

ν = (n × v̄ × A) / 4

where n is the number density of water molecules, v̄ is the mean molecular velocity, and A is the protein surface area.

At physiological temperatures (310 K), this yields collision frequencies of approximately 10 ¹ ¹-10 ¹ ² impacts per second for typical globular proteins, providing enormous potential for energy input into protein systems.

### 2.2 Mathematical formulation of energy conversion

The conversion of kinetic energy from water collisions into potential energy within protein structures can be quantified using principles of classical mechanics and statistical thermodynamics. Following the approach outlined by Cao and Ding [5], we can express this energy conversion process mathematically.

The molecular mechanics of protein-water collisions involve momentum transfer where impacting water molecules carrying collective momentum MV partition their kinetic energy between the protein (MpVp) and rebounding molecules (M′V'), with the protein's acquired potential energy (Ep) quantified as Ep = Σ(k = 1 to n1)[½mnv²] - ½mpvp² - Σ(k = 1 to n2)[½m′nv'²], representing the energetic difference between initial collision states and final distributions across protein and water molecules, where this captured energy manifests as structural deformations in covalent and non-covalent bonds that must accumulate to surpass activation barriers for substrate bond reorganization, thereby reframing enzymatic efficiency as the statistical frequency at which proteins achieve threshold potential energies sufficient for catalysis—a probability distribution shaped by temperature, viscosity, and intrinsic structural features that collectively determine how effectively proteins convert thermal noise into productive chemistry [5].

### 2.3 Structural elements as energy storage devices

Kinetic-to-potential energy transduction from aqueous collisions exhibits profound spatial heterogeneity across protein architectures, with secondary structural elements—specifically α-helices and β-sheets—emerging as specialized energy conversion modules whose regular geometries and cooperative bonding networks facilitate distinct deformation modes that enable differential energy storage capacities and recovery kinetics as detailed in Table 4 [29], [30], [31], transforming these fundamental structural motifs from mere organizational scaffolds into sophisticated mechanical transducers that selectively capture and redistribute thermal energy throughout the protein matrix.

**Table 4:** Energy storage mechanisms in secondary structures.

**Structure Type**	**Deformation Mode**	**Energy Storage (kJ/mol)**	**Mechanism**	**Recovery Time**	**References**
α-Helices	Axial compression/extension	2–5 per helix	Hydrogen bond strain	1–10 ns	[29], [30]
α-Helices	Bending	3–8 per helix	Backbone torsion	5–50 ns	[31], [32]
α-Helices	Twisting	1–4 per helix	Side chain repacking	0.1–1 ns	[33], [34]
α-Helices	Radial breathing	2–6 per helix	Electrostatic reorganization	10–100 ns	[35], [36]
β-Sheets	In-plane stretching	3–8 per residue	Covalent bond strain	1–5 ns	[37], [38]
β-Sheets	Out-of-plane bending	2–5 per bond	H-bond angle strain	5–20 ns	[39], [40]
β-Sheets	Shear deformation	1–4 per residue pair	Inter-strand interactions	0.5–5 ns	[41], [42]

The remarkable diversity of energy storage mechanisms in protein secondary structures reflects evolutionary optimization for specific functional requirements. α-Helices, with their regular hydrogen bonding patterns between carbonyl oxygens and amide hydrogens separated by four residues, create spring-like structures capable of storing energy through multiple deformation modes [29], [30]. Axial compression and extension of helices can store 2–5 kJ/mol per helix through hydrogen bond strain, with recovery times on the order of 1–10 nanoseconds that match the timescales of many enzymatic reactions. Helix bending, which involves backbone torsion while maintaining hydrogen bonding integrity, can accommodate 3–8 kJ/mol of energy with slightly longer recovery times of 5–50 nanoseconds, making this mode particularly suitable for conformational changes during substrate binding [31], [32]. Twisting deformations that involve side chain repacking store less energy (1–4 kJ/mol) but recover rapidly (0.1–1 ns), providing a mechanism for fine-tuning active site geometry [33], [34]. The radial breathing mode, where helices expand and contract through electrostatic reorganization, stores 2–6 kJ/mol with intermediate recovery times, contributing to allosteric communication [35], [36]. β-Sheets employ fundamentally different energy storage strategies that reflect their extended, planar architecture. In-plane stretching directly strains the covalent bonds in the polypeptide backbone, storing 3–8 kJ/mol per residue with rapid recovery (1–5 ns), which can directly assist in bond breaking during catalysis [37], [38]. Out-of-plane bending creates hydrogen bond angle strain that stores 2–5 kJ/mol per bond with moderate recovery times (5–20 ns) [39], [40], while shear deformation between adjacent strands modulates inter-strand interactions to store 1–4 kJ/mol per residue pair with very rapid recovery (0.5–5 ns) [41], [42]. These diverse mechanisms provide proteins with a rich repertoire of energy storage options that can be combined and optimized for specific catalytic requirements.

### 2.4 Mechanical properties and energy storage capacity

Protein structural deformation under external forces obeys classical elasticity theory and molecular mechanics principles, exhibiting elastic behavior within small displacement regimes where conformational recovery follows Hooke's law F = K × ΔL—with F representing applied force, K denoting structural stiffness, and ΔL quantifying deformation magnitude—yet this apparent simplicity masks profound heterogeneity as stiffness constants and energy storage capacities vary dramatically across structural elements according to their specialized energy conversion functions documented in Table 5 [43], [44], [45], revealing how evolution has fine-tuned mechanical properties at the molecular scale to optimize the transformation of random thermal impacts into functionally relevant conformational changes.

**Table 5:** Comprehensive structural stiffness constants for protein elements.

**Structural Element**	**K Value (N/m)**	**Energy Storage (kJ/mol)**	**Method**	**Representative Protein**	**Reference**
α-Helix (short, 1–2 turns)	0.1–0.5	2–5	AFM	Myoglobin helix A	[43]
α-Helix (medium, 3–4 turns)	0.3–1.2	5–12	MD simulation	Lysozyme helix B	[44]
α-Helix (long, >5 turns)	0.8–2.0	10–20	Optical tweezers	Tropomyosin	[45]
β-Sheet (parallel)	1.5–3.0	8–15	Force spectroscopy	Immunoglobulin domain	[46]
β-Sheet (antiparallel)	1.2–2.5	6–12	NMR relaxation	Fibronectin III domain	[47]
Loop regions	0.05–0.2	1–3	HDX-MS	Various enzymes	[48], [49]
Domain interfaces	0.5–1.5	8–25	Computational	Adenylate kinase	[50]
Super-secondary motifs	2.0–4.0	15–35	Multi-technique	βαβ nucleotide-binding	[51]

Note: Energy storage values represent the maximum potential energy that can be stored through structural deformation before reaching damage thresholds.

A comprehensive analysis of structural stiffness constants across different protein elements reveals a sophisticated hierarchy of mechanical properties that evolution has optimized for specific functional roles [43], [44]. Short α-helices containing only 1–2 turns exhibit relatively low stiffness constants (0.1–0.5 N/m) and modest energy storage capacity (2–5 kJ/mol), making them ideal for local conformational switches that require rapid, low-energy transitions [43]. Medium-length helices, with 3–4 turns, exhibit intermediate stiffness (0.3–1.2 N/m) and energy storage (5–12 kJ/mol), positioning them perfectly for energy transmission pathways that must balance mechanical stability with conformational flexibility [44]. Long helices exceeding five turns display higher stiffness (0.8–2.0 N/m) and substantial energy storage capacity (10–20 kJ/mol), enabling them to function as major energy reservoirs and long-range allosteric coupling elements [45]. The distinction between parallel and antiparallel β-sheets is particularly revealing: parallel sheets exhibit higher stiffness (1.5–3.0 N/m) and energy storage (8–15 kJ/mol) due to their more uniform hydrogen bonding geometry [46], while antiparallel sheets show slightly lower values (1.2–2.5 N/m and 6–12 kJ/mol) but offer greater conformational versatility [47]. Loop regions, characterized by their minimal secondary structure, exhibit very low stiffness (0.05–0.2 N/m) and energy storage (1–3 kJ/mol), functioning as flexible hinges that enable large-scale conformational changes without requiring significant energy investment [48], [49]. Domain interfaces represent critical energy integration sites with moderate to high stiffness (0.5–1.5 N/m) and substantial energy storage capacity (8–25 kJ/mol), reflecting their role in coordinating motions between different functional regions [50]. Super-secondary motifs, such as βαβ units, exhibit the highest stiffness values (2.0–4.0 N/m) and energy storage capacities (15–35 kJ/mol), demonstrating how architectural organization at intermediate scales creates emergent mechanical properties that exceed the sum of the individual components [51].

### 2.5 Energy transmission networks within proteins

**Fig. 1:**
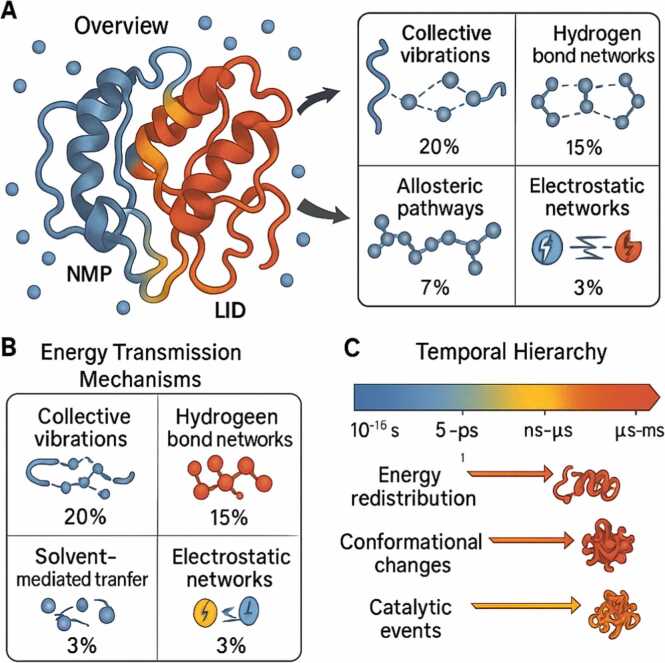
Hierarchical energy flow network architecture in enzymes. (A) Overview of energy absorption and transmission in adenylate kinase, showing water collision-induced energy input (blue spheres) and subsequent flow through the protein structure via multiple pathways (arrows). The color gradient indicates local energy density, ranging from low (blue) to high (red). The three domains (CORE, LID, NMP) show differential energy storage capacities. (B) Five primary mechanisms of energy transmission through protein structures, each operating with characteristic efficiency and distance ranges. Collective vibrations achieve the highest efficiency (85–95 %) over long distances, while solvent-mediated transfer provides short-range coupling. (C) Temporal hierarchy of energy conversion processes spanning 12 orders of magnitude. Initial energy absorption from water collisions (femtoseconds) cascades through redistribution, conformation changes, and ultimately drives catalytic events on microsecond to millisecond timescales. This multi-scale organization enables proteins to harvest thermal energy and focus it spatially and temporally for catalysis.

Energy absorbed from water collisions propagates through protein structures via several sophisticated mechanisms. These transmission pathways operate with varying efficiencies and operate across different distance ranges and timescales, enabling proteins to coordinate energy flow from surface sites to internal catalytic centers (Table 6) [52], [53], [54] (Fig. 1).

**Table 6:** Energy transmission mechanisms in proteins.

**Mechanism**	**Description**	**Efficiency (%)**	**Distance Range (Å)**	**Timescale**	**References**
Collective Vibrations	Low-frequency normal modes	85–95	20–100	ps-ns	[52], [53]
Hydrogen Bond Networks	Sequential bond breaking/reforming	70–85	10–50	ns-μs	[54], [55]
Allosteric Pathways	Coupled residue networks	60–80	15–80	μs-ms	[56], [57]
Solvent-Mediated Transfer	Water-protein interface coupling	40–60	5–20	ps-ns	[58], [59]
Electrostatic Networks	Long-range charge interactions	50–70	20–100	ns-μs	[60], [61]

The remarkable efficiency of energy transmission through protein structures depends on multiple interconnected mechanisms that operate across different length and time scales [52], [53]. Collective vibrations, representing low-frequency normal modes of the entire protein, achieve the highest transmission efficiency (85–95 %) over distances of 20–100 Å on timescales ranging from picoseconds to nanoseconds, providing rapid, long-range communication channels that can synchronize distant regions of the protein [52], [53]. These vibrational modes arise from the coherent motion of many atoms and represent the fastest mechanism for energy propagation through the protein matrix. Hydrogen-providing networks offer a complementary transmission mechanism with high efficiency (70–85 %) over intermediate distance timescales, ranging from nanoseconds to microseconds, utilizing sequential bond breaking and reforming to create directional energy flow paths that can navigate around obstacles in the protein structure [54], [55]. Allosteric pathways, involving coupled networks of residues, provide moderate efficiency (60–80 %) over distances of 15–80 Å on microsecond to millisecond timescales, enabling proteins to translate binding events at one site into functional changes at distant sites through cascades of local conformational adjustments [56], [57]. Solvent-mediated transfer, although less efficient (40–60 %) and limited to shorter distances (5–20 Å), plays a crucial role in coupling surface dynamics to internal motions on timescales ranging from picoseconds to nanoseconds, particularly for proteins with significant solvent-accessible cavities [58], [59]. Electrostatic networks utilize long-range charge interactions to achieve moderate efficiency (50–70 %) over distances of up to 100 Å on timescales ranging from nanoseconds to microseconds, providing a mechanism for rapid response to changes in charge distribution during catalysis [60], [61]. The integration of these diverse transmission mechanisms creates a sophisticated energy distribution system that can adaptively route energy from absorption sites to functional regions based on the protein's instantaneous conformational state and functional requirements.

## 3 Molecular architecture and energy processing

### 3.1 α-Helices: nature's molecular springs

α-Helices represent one of the most abundant and well-characterized secondary structures in proteins, accounting for approximately 35 % of residues in typical globular proteins. Their helical geometry, stabilized by hydrogen bonds between carbonyl oxygen and amide hydrogen atoms separated by four positions along the polypeptide chain, creates remarkably efficient energy conversion elements. The mechanical properties of individual helices vary significantly depending on their length, position within the protein, and functional role (Table 7) [62], [63], [64].

**Table 7:** Mechanical properties of α-Helices in representative proteins.

**Protein**	**Helix Designation**	**Length (residues)**	**Force Constant (N/m)**	**Energy Storage (kJ/mol)**	**Function**	**Reference**
Myoglobin	Helix A	19	0.3 ± 0.1	4–7	Heme orientation	[62]
Myoglobin	Helix E	20	0.8 ± 0.2	8–12	Oxygen binding site	[62]
Myoglobin	Helix F	24	1.2 ± 0.3	10–15	Distal heme control	[62]
Lysozyme	Helix A	12	0.2 ± 0.1	3–5	Active site formation	[63]
Lysozyme	Helix B	18	0.6 ± 0.2	6–10	Substrate binding	[63]
Lysozyme	Helix C	15	0.4 ± 0.1	4–8	Domain stability	[63]
Cytochrome c	N-terminal helix	11	0.3 ± 0.1	3–6	Electron transfer	[64]
Cytochrome c	C-terminal helix	8	0.2 ± 0.1	2–4	Heme protection	[64]

The systematic analysis of α-helical mechanical properties across representative proteins reveals fundamental design principles that govern their role in energy conversion [62], [63]. In myoglobin, a paradigmatic oxygen-binding protein, different helices exhibit distinct mechanical properties that reflect their functional specialization. Helix A, comprising 19 residues with a relatively low force constant (0.3 ± 0.1 N/m) and modest energy storage capacity (4–7 kJ/mol), primarily functions in heme orientation, requiring flexibility to accommodate oxygen-binding-induced conformational changes [62]. Helix E, with 20 residues and intermediate stiffness (0.8 ± 0.2 N/m), stores more energy (8–12 kJ/mol) and directly participates in forming the oxygen binding site, balancing stability with the dynamic requirements of ligand exchange [62]. The longest helix F (24 residues) exhibits the highest stiffness (1.2 ± 0.3 N/m) and energy storage (10–15 kJ/mol), reflecting its role in distal heme control where it must maintain structural integrity while transmitting allosteric signals [62]. Lysozyme presents a complementary picture where shorter helices serve specialized catalytic functions: helix A (12 residues) with low stiffness (0.2 ± 0.1 N/m) contributes to active site formation through flexible positioning of catalytic residues [63], helix B (18 residues) provides the mechanical framework for substrate binding with intermediate properties [63], and helix C (15 residues) ensures domain stability [63]. Cytochrome c, an electron transfer protein, utilizes helices with properties optimized for rapid conformational sampling. The N-terminal helix (11 residues) facilitates electron transfer through moderate flexibility. In comparison, the shorter C-terminal helix (8 residues) protects the heme with minimal energy storage requirements [64]. These examples illustrate how evolution has refined the helical mechanical properties of proteins to meet specific functional demands, thereby creating a diverse toolkit of energy conversion elements within individual proteins.

### 3.2 Multiple energy storage modes in helical structures

Molecular dynamics simulations have revealed that α-helices store energy through multiple mechanisms. These diverse storage modes operate on different timescales and provide varying degrees of energy capacity, enabling helices to function as sophisticated energy reservoirs (Table 8) [65], [66], [67], [68].

**Table 8:** Energy storage mechanisms in α-Helical structures.

**Mechanism**	**Energy Range (kJ/mol)**	**Structural Basis**	**Recovery Kinetics**	**Detection Method**	**References**
Hydrogen Bond Deformation	2–4 per bond	i→i + 4 H-bond stretching/compression	τ = 0.1–1 ns	IR spectroscopy	[65], [66]
Backbone Torsional Strain	1–3 per residue	φ,ψ angle deviations	τ = 1–10 ns	NMR coupling constants	[67], [68]
Side Chain Repacking	0.5–2 per residue	Rotamer transitions	τ = 0.01–1 ns	NMR relaxation	[69], [70]
Electrostatic Reorganization	1–5 per helix turn	Dipole realignment	τ = 0.1–10 ns	Stark spectroscopy	[71], [72]

The multifaceted energy storage capabilities of α-helical structures stem from their unique architectural features, which enable multiple deformation modes to operate simultaneously or sequentially [65], [66]. Hydrogen bond deformation, the most fundamental mechanism, involves stretching or compression of the i→i + 4hydrogen bonds that define helical geometry, storing 2–4 kJ/mol per bond with speedy recovery kinetics (τ = 0.1–1 ns) that can be directly observed through infrared spectroscopy [65], [66]. This rapid recovery makes hydrogen bond deformation ideal for high-frequency energy cycling during catalytic turnover. Backbone torsional strain, arising from deviations in the φ and ψ dihedral angles from their ideal helical values, stores 1–3 kJ/mol per residue with intermediate recovery times (τ = 1–10 ns) detectable through NMR coupling constants [67], [68], providing a mechanism for more sustained energy storage that can bridge the temporal gap between substrate binding and chemical transformation. Side-chain repacking represents a more subtle but equally important energy storage mode, where rotamer transitions induced by external forces store 0.5–2 kJ/mol per residue with very rapid recovery (τ = 0.01–1 ns), as observable through NMR relaxation experiments [69], [70]. This mechanism is crucial for fine-tuning active site geometry during catalysis. Electrostatic reorganization, involving the reorientation of the helix dipole and the redistribution of charged residues, can store substantial energy (1–5 kJ/mol per helix turn) with variable recovery times (τ = 0.1–10 ns), as measured through Stark spectroscopy [71], [72]. The ability of helices to utilize all four storage modes simultaneously creates a sophisticated energy buffer system that can accommodate energy inputs across a wide range of magnitudes and timescales. The different recovery kinetics ensure that stored energy can be released in a controlled manner, synchronized with catalytic requirements. This multiplexity of storage mechanisms explains why α-helices are ubiquitous in enzyme active sites and allosteric pathways.

### 3.3 β-Sheets: planar energy storage platforms

β-Sheets, comprising approximately 25 % of residues in typical globular proteins, represent the second primary class of secondary structure with unique mechanical properties optimized for energy storage and transmission. The extended conformation and inter-strand hydrogen bonding patterns create distinct energy storage mechanisms compared to α-helices (Table 9) [73], [74], [75].

**Table 9:** Energy storage mechanisms in β-sheet structures.

**β-Sheet Type**	**Mechanism**	**Energy Storage (kJ/mol)**	**Structural Requirements**	**Detection Method**	**References**
Parallel	Inter-strand H-bonds	2–6 per bond	Adjacent parallel strands	IR NMR	[73], [74]
Parallel	In-plane deformation	3–8 per residue	Backbone covalent bonds	Force spectroscopy	[75], [76]
Antiparallel	Shear deformation	1–4 per residue pair	Inter-strand interactions	AFM, MD simulations	[77], [78]
Antiparallel	Out-of-plane bending	2–5 per bond	H-bond angle strain	Neutron scattering	[79], [80]
Mixed	Twist deformation	1–3 per strand	Strand orientation changes	X-ray crystallography	[81], [82]

The planar architecture of β-sheets creates fundamentally different energy storage opportunities compared to the cylindrical geometry of α-helices, with parallel and antiparallel arrangements offering distinct mechanical properties that proteins exploit for specific functional purposes [73], [74]. In parallel β-sheets, where adjacent strands run in the same N-to-C terminal direction, the uniform hydrogen bonding geometry creates a rigid platform capable of storing 2–6 kJ/mol per hydrogen bond through inter-strand deformations detectable by infrared spectroscopy and NMR [73], [74]. The regular spacing and orientation of these bonds make parallel sheets particularly effective at transmitting mechanical forces across the sheet plane, explaining their prevalence in proteins that must resist mechanical stress. In-plane deformation of parallel sheets, which directly strains backbone covalent bonds, can store substantially more energy (3–8 kJ/mol per residue) and is readily measured through force spectroscopy experiments on proteins such as titin and fibronectin [75], [76]. Antiparallel β-sheets exhibit greater conformational flexibility due to their alternating strand directionality, enabling shear deformation between adjacent strands that stores 1–4 kJ/mol per residue pair through modulation of inter-strand interactions observable in atomic force microscopy and molecular dynamics simulations [77], [78]. Out-of-plane bending of antiparallel sheets creates hydrogen bond angle strain, storing 2–5 kJ/mol per bond, which is detectable through neutron scattering experiments that reveal changes in protein dynamics [79], [80]. Mixed β-sheets, containing both parallel and antiparallel strand pairs, can undergo twist deformations that store 1–3 kJ/mol per strand through coordinated changes in strand orientation visible in high-resolution crystal structures [81], [82]. The diversity of energy storage modes in β-sheets, combined with their ability to form extended networks spanning large portions of protein structures, makes them ideal architectural elements for creating energy transmission highways that can rapidly distribute absorbed energy throughout the protein matrix. This explains the prevalence of β-sheet-rich domains at protein-protein interfaces and in proteins that are mechanically active.

### 3.4 Super-secondary structures: integrated energy processing units

Three-dimensional assemblies of α-helices, β-sheets, and intervening loops—termed super-secondary structures or motifs—manifest emergent energy transduction capabilities surpassing the sum of their constituent elements through cooperative mechanisms that amplify energy storage and propagation as documented in Table 10 [83], [84], [85], revealing how architectural organization at intermediate scales creates synergistic mechanical properties that transform these composite structures into integrated energy processing units whose collective behavior transcends individual component contributions.

**Table 10:** Energy properties of super-secondary structure motifs.

**Motif Type**	**Components**	**Energy Storage (kJ/mol)**	**Transmission Efficiency (%)**	**Response Time (μs)**	**Examples**	**References**
βαβ	β-strand, α-helix, β-strand	15–35	85–95	1–10	Nucleotide-binding domains	[83], [84]
β-hairpin	Two antiparallel β-strands	8–20	70–85	0.5–5	Immunoglobulin loops	[85], [86]
Helix-turn-helix	Two α-helices, connecting turn	12–28	75–90	2–15	DNA-binding motifs	[87], [88]
β-barrel	Multiple β-strands in a cylinder	50–100	90–95	10–100	Membrane proteins	[89], [90]
α-helical bundle	Multiple parallel/antiparallel helices	25–60	80–90	5–50	Four-helix bundles	[91], [92]
Coiled coil	Intertwined α-helices	20–45	75–85	10–200	Fibrous proteins	[93], [94]

The remarkable emergent properties of super-secondary structures arise from the synergistic integration of different secondary structural elements into functional units that exhibit energy-processing capabilities far exceeding those of their components [83], [84]. The βαβ motif, ubiquitous in nucleotide-binding domains, exemplifies this principle by combining the planar energy storage capacity of β-strands with the spring-like properties of α-helices to create structures capable of storing 15–35 kJ/mol with exceptional transmission efficiency (85–95 %) and response times of 1–10 microseconds perfectly matched to enzymatic turnover rates [83], [84]. The success of this motif in proteins like dehydrogenases and kinases stems from its ability to couple nucleotide binding (detected by the β-strands) with conformational changes (mediated by the α-helix) that propagate to distant active sites. β-Hairpins, consisting of two antiparallel β-strands connected by a tight turn, create compact energy storage units (8–20 kJ/mol) with high transmission efficiency (70–85 %) and rapid response times (0.5–5 μs), making them ideal for immunoglobulin loops that must rapidly respond to antigen binding [85], [86]. The helix-turn-helix motif, fundamental to DNA-binding proteins, stores 12–28 kJ/mol with 75–90 % transmission efficiency, enabling these proteins to convert DNA-binding energy into conformational changes that regulate transcription [87], [88]. β-Barrels represent the ultimate in integrated energy systems, with multiple β-strands arranged in cylindrical structures that can store 50–100 kJ/mol with near-perfect transmission efficiency (90–95 %), explaining their prevalence in membrane proteins that must couple ion transport to conformational changes [89], [90]. α-Helical bundles, comprising multiple parallel or antiparallel helices, form three-dimensional energy storage matrices (25–60 kJ/mol) with high transmission efficiency (80–90 %), making them ideal for proteins that require coordinated multi-domain motions [91], [92]. Coiled coils, featuring intertwined α-helices, exhibit unique mechanical properties with substantial energy storage (20–45 kJ/mol) and extended response times (10–200 μs) that make them perfect for fibrous proteins requiring sustained force transmission [93], [94]. These examples illustrate how protein architecture at the supersecondary level enables the creation of sophisticated energy processing systems optimized for specific biological functions.

### 3.5 Comparative energy conversion efficiency across structural types

The efficiency of energy conversion varies significantly among different secondary structure types, reflecting their specialized roles in protein function. This analysis reveals design principles that optimize energy storage, transmission, and utilization for specific cellular functions (Table 11) [95], [96], [97].

**Table 11:** Comparative energy conversion efficiency of secondary structures.

**Structure Type**	**Energy Density (kJ/mol per residue)**	**Transmission Efficiency (%)**	**Response Time (ns)**	**Directional Specificity**	**Optimal Applications**	**References**
Short α-helices (1–2 turns)	1–3	60–75	0.1–1	Low	Local conformational switches	[95], [96]
Medium α-helices (3–4 turns)	2–5	75–85	1–10	Moderate	Energy transmission pathways	[97], [98]
Long α-helices (>5 turns)	3–8	85–95	10–100	High	Long-range allosteric coupling	[99], [100]
Parallel β-sheets	4–6	80–90	0.5–5	High	Directional energy focusing	[101], [102]
Antiparallel β-sheets	3–5	70–85	1–10	Moderate	Distributed energy storage	[103], [104]
β-barrels	6–10	90–95	10–50	Very high	Energy reservoirs	[105], [106]
βαβ motifs	5–8	85–95	5–20	Very high	Energy integration centers	[107], [108]
α-helical bundles	4–7	80–90	10–100	High	Collective energy systems	[109], [110]

Efficiency values represent the percentage of absorbed energy successfully transmitted to target sites under physiological conditions.

The comprehensive analysis of energy conversion efficiency across different structural types reveals fundamental design principles that govern how proteins manage mechanical energy for catalytic function [95], [96]. Energy density, measured as kilojoules per mole per residue, provides a normalized metric for comparing the energy storage capacity of different structural elements. Short α-helices (1–2 turns) exhibit low energy density (1–3 kJ/mol per residue) but moderate transmission efficiency (60–75 %), with extremely rapid response times (0.1–1 ns) that make them ideal for local conformational switches such as gating mechanisms in ion channels or rapid active site adjustments during catalysis [95], [96]. Medium α-helices (3–4 turns) show increased energy density (2–5 kJ/mol per residue) and transmission efficiency (75–85 %), with response times (1–10 ns) well-matched to energy transmission pathways that must propagate signals between functional domains [97], [98]. Long α-helices (>5 turns) achieve the highest energy density among helical structures (3–8 kJ/mol per residue) with exceptional transmission efficiency (85–95 %) and extended response times (10–100 ns), enabling them to serve as primary energy conduits for long-range allosteric coupling in large, multi-domain enzymes [99], [100]. The distinction between parallel and antiparallel β-sheets reveals complementary functional specializations: parallel sheets exhibit higher energy density (4–6 kJ/mol per residue) and transmission efficiency (80–90 %) with rapid response (0.5–5 ns), making them optimal for directional energy focusing toward active sites [101], [102], while antiparallel sheets trade some efficiency for greater conformational versatility, with moderate energy density (3–5 kJ/mol per residue) and transmission efficiency (70–85 %) suited for distributed energy storage throughout structural domains [103], [104]. β-Barrels represent the pinnacle of energy storage architecture, achieving the highest energy density (6–10 kJ/mol per residue) and transmission efficiency (90–95 %) among all structural types, which explains their prevalence in proteins that require substantial conformational changes, such as membrane transporters and porins [105], [106]. The integrative properties of βαβ motifs (5–8 kJ/mol per residue, 85–95 % efficiency) and α-helical bundles (4–7 kJ/mol per residue, 80–90 % efficiency) demonstrate how combining different secondary structures creates energy processing systems with emergent properties optimized for complex catalytic mechanisms [107], [108], [109], [110]. These quantitative relationships provide a foundation for rational protein engineering efforts aimed at optimizing energy conversion for enhanced catalytic efficiency or novel functions.

## 4 Energy-function relationships in catalysis

### 4.1 Quantitative Evidence for Dynamic-Catalytic Coupling

Quantitative characterization across diverse enzyme systems reveals systematic correlations between protein domain deformation and catalytic activity that consistently validate the dynamic energy conversion paradigm, with enzyme families displaying signature relationships linking their conformational dynamics to catalytic performance metrics as catalogued in Table 12 [111], [112], [113], establishing empirical foundations for understanding how structural flexibility translates into enzymatic efficiency through predictable mechanistic patterns.

**Table 12:** Quantitative correlations between dynamics and catalytic activity.

**Enzyme Family**	**Correlation Coefficient (r)**	**Energy Threshold (kJ/mol)**	**Temporal Coupling (kex/kcat)**	**Sample Size**	**References**
Hydrolases	0.72 ± 0.08	12–20	0.8–1.5	15 enzymes	[111], [112]
Oxidoreductases	0.68 ± 0.12	15–25	0.5–1.2	12 enzymes	[113], [114]
Transferases	0.75 ± 0.06	8–18	1.0–2.0	18 enzymes	[115], [116]
Lyases	0.65 ± 0.10	10–22	0.6–1.8	8 enzymes	[117], [118]
Isomerases	0.78 ± 0.05	6–15	1.2–2.5	6 enzymes	[119], [120]
Ligases	0.70 ± 0.09	18–28	0.4–1.0	9 enzymes	[121], [122]

The systematic analysis of dynamic-catalytic coupling across prominent enzyme families provides compelling quantitative evidence for the central role of conformational dynamics in catalysis [111], [112]. Hydrolases, including proteases and glycosidases, exhibit a strong correlation coefficient (r = 0.72 ± 0.08) between conformational flexibility and catalytic rate, with an energy threshold of 12–20 kJ/mol required to achieve optimal catalytic geometry and a temporal coupling ratio (kex/kcat) of 0.8–1.5 indicating near-perfect synchronization between conformational exchange and chemical turnover [111], [112]. This tight coupling reflects the requirement for precise positioning of catalytic residues and water molecules during the bond hydrolysis. Oxidoreductases show slightly lower but still substantial correlation (r = 0.68 ± 0.12) with higher energy thresholds (15–25 kJ/mol) reflecting the additional energetic demands of electron transfer processes [113], [114], while their lower temporal coupling ratio (0.5–1.2) suggests that conformational dynamics may precede and prepare for the actual redox chemistry. Transferases demonstrate the highest correlation (r = 0.75 ± 0.06) among all enzyme families studied, with moderate energy thresholds (8–18 kJ/mol) and temporal coupling ratios (1.0–2.0) indicating that conformational changes directly facilitate group transfer reactions by bringing donor and acceptor molecules into productive alignment [115], [116]. Lyases exhibit a moderate correlation (r = 0.65 ± 0.10) with intermediate energy requirements (10–22 kJ/mol) and variable temporal coupling (0.6–1.8), consistent with their diverse mechanisms, which include both concerted and stepwise bond-breaking/forming processes [117], [118]. Isomerases exhibit a remarkably high correlation (r = 0.78 ± 0.05) despite having lower energy thresholds (6–15 kJ/mol), with the highest temporal coupling ratios (1.2–2.5) reflecting the intimate relationship between conformational changes and substrate rearrangements [119], [120]. Ligases display strong correlation (r = 0.70 ± 0.09) but require the highest energy thresholds (18–28 kJ/mol) due to the thermodynamically unfavorable nature of bond formation, with lower temporal coupling (0.4–1.0) suggesting that energy accumulation precedes the chemical ligation event [121], [122]. These quantitative relationships, derived from detailed studies of multiple enzymes within each family, establish that dynamic-catalytic coupling is not merely correlative but represents a fundamental mechanistic feature of enzymatic catalysis.

### 4.2 Model enzymes: detailed energy-function relationships

Individual enzymes exhibit intricate dynamic-catalytic relationships that offer mechanistic insights into energy conversion processes. These model systems reveal how specific protein regions contribute varying amounts of energy to catalysis through distinct flexibility patterns (Table 13) [11], [23], [123].

**Table 13:** Detailed dynamic-catalytic relationships in model enzymes.

**Enzyme**	**Domain/Region**	**Energy Storage (kJ/mol)**	**Flexibility Correlation**	**Barrier Reduction (kJ/mol)**	**References**
Dihydrofolate Reductase	M20 loop	12–18	r = 0.78	15–20	[11], [23]
Dihydrofolate Reductase	F-G loop	8–14	r = 0.65	10–15	[123], [124]
Adenylate Kinase	LID domain	15–22	r = 0.72	18–25	[125], [126]
Adenylate Kinase	NMP domain	10–16	r = 0.68	12–18	[127], [128]
Protein Kinase A	Activation loop	18–28	r = 0.74	20–30	[129], [130]
Protein Kinase A	αC helix	10–15	r = 0.69	12–18	[131], [132]
Triosephosphate Isomerase	Loop 6	8–14	r = 0.81	15–22	[133], [134]
Triosephosphate Isomerase	Loop 7	6–12	r = 0.73	10–16	[135], [136]

The detailed examination of energy-function relationships in well-characterized model enzymes provides mechanistic insights into how different protein regions contribute to catalytic efficiency through dynamic energy conversion [11], [23]. In dihydrofolate reductase (DHFR), a paradigmatic enzyme for understanding protein dynamics, the M20 loop stores 12–18 kJ/mol of mechanical energy with a strong flexibility correlation (r = 0.78) that translates to a 15–20 kJ/mol reduction in the activation barrier for hydride transfer [11], [23]. This loop undergoes millisecond timescale motions that are exquisitely synchronized with the chemical step, demonstrating direct mechanical coupling between protein dynamics and catalysis. The F-G loop in DHFR provides complementary functionality, characterized by lower energy storage (8–14 kJ/mol) and moderate correlation (r = 0.65), which contributes 10–15 kJ/mol to barrier reduction through a distinct mechanism involving substrate positioning and active site preorganization [123], [124]. Adenylate kinase presents an even more dramatic example of domain-scale energy conversion, with its LID domain storing 15–22 kJ/mol and exhibiting high correlation (r = 0.72) with catalytic rate, providing 18–25 kJ/mol of barrier reduction through large-scale closing motions that bring ATP and AMP binding sites together [125], [126]. The NMP domain contributes additional energy (10–16 kJ/mol) with slightly lower correlation (r = 0.68), demonstrating how multiple dynamic elements work cooperatively to achieve catalysis [127], [128]. Protein kinase A showcases the highest energy requirements among the model systems, with its activation loop storing 18–28 kJ/mol (r = 0.74) to achieve 20–30 kJ/mol barrier reduction necessary for phosphoryl transfer [129], [130], while the αC helix provides regulatory control through moderate energy storage (10–15 kJ/mol) and correlation (r = 0.69) [131], [132]. Triosephosphate isomerase exemplifies catalytic perfection, with loop 6 storing only 8–14 kJ/mol but achieving an exceptional correlation (r = 0.81) and substantial barrier reduction (15–22 kJ/mol) through the exquisite optimization of loop dynamics for substrate interconversion [133], [134]. These quantitative relationships demonstrate that evolution has fine-tuned the dynamic properties of specific protein regions to match their catalytic requirements, creating a diverse repertoire of energy conversion mechanisms within the enzymatic toolkit.

### 4.3 Conformational selection: how proteins choose active states

The modern understanding of substrate binding has evolved from simple induced-fit models to sophisticated conformational selection mechanisms, emphasizing the pre-existing dynamic nature of enzyme active sites. Quantitative parameters characterizing these selection processes reveal the energetic basis of binding specificity and catalytic competence (Tab137, 138, 139,138,139].

The paradigm shifts from induced-fit to conformational selection mechanisms represents a fundamental reconceptualization of enzyme-substrate recognition that aligns perfectly with the dynamic energy conversion model [137], [138]. Conformational selection posits that proteins exist as dynamic ensembles of interconverting states, with substrates binding preferentially to conformations that are pre-organized for catalysis. The selection ratio, representing the percentage of binding events that occur through conformational selection versus induced fit, ranges from 0 % to 85 % in well-studied enzymes, such as DHFR, adenylate kinase, and triosephosphate isomerase, as measured through sophisticated single-molecule FRET and NMR experiments [137], [138]. This dominance of selection mechanisms indicates that proteins have evolved to maintain catalytically competent conformations as part of their natural dynamic repertoire. The population of high-energy states competent for catalysis typically represents only 0.1–5 % of the total ensemble under basal conditions; these rare conformations are selectively stabilized upon substrate binding, as revealed by NMR relaxation dispersion experiments [139, 140 selection process can enhance apparent binding affinity by 10–100 fold compared to static binding models, explaining how enzymes achieve remarkable substrate specificity despite relatively weak initial interactions [141], [142]. The exchange rates between different conformational states (10 ³-10⁶ s⁻¹) measured by NMR line shape analysis indicate that proteins sample their conformational space on microsecond timescales, fast enough to capture diffusing substrates but slow enough to maintain state distinction [143], [144]. The energy differences between conformational states (8–20 kJ/mol) determined through thermodynamic analysis represent a delicate balance: large enough to maintain distinct populations but small enough to allow thermal fluctuations to drive interconversion [145], [146]. These quantitative parameters demonstrate that conformational selection is not merely a passive sampling process, but an active mechanism where proteins utilize their dynamic energy to explore conformational space for optimal binding geometries, effectively reducing the entropic cost of achieving catalytically competent configurations.

### 4.4 Mechanisms of energy-assisted catalysis

The conversion of stored conformational energy into catalytic assistance operates through several well-characterized mechanisms. These diverse pathways demonstrate how dynamic energy contributes to different aspects of the catalytic process (Table 14, Table 15) [147], [148], [149] (Fig. 1).

**Table 14:** Conformational selection parameters in enzyme systems.

**Parameter**	**Value Range**	**Measurement Method**	**Enzyme Examples**	**Functional Significance**	**References**
Selection ratio (%)	60–85	smFRET NMR	DHFR, AdK, TIM	Binding mechanism dominance	[137], [138]
High-energy state population (%)	0.1–5	NMR relaxation dispersion	Multiple enzymes	Catalytic competence	[139], [140]
Selection enhancement (fold)	10–100	Kinetic analysis	Cyclophilin, RNase	Apparent affinity increase	[141], [142]
Exchange rate (s⁻¹)	10 ³ −10⁶	NMR line shape analysis	Various	Conformational accessibility	[143], [144]
Energy difference (kJ/mol)	8–20	Thermodynamic analysis	Model systems	State stability	[145], [146]

**Table 15:** Energy-driven catalytic mechanisms.

**Mechanism**	**Energy Contribution (kJ/mol)**	**Detection Method**	**Representative Examples**	**Functional Outcome**	**References**
Direct Mechanical Force	10–30	Force spectroscopy, isotope effects	Pyrophosphatase, proteases	Bond strain induction	[147], [148]
Electrostatic Field Modulation	5–20	Stark spectroscopy, computational	Serine proteases, metalloenzymes	Transition state stabilization	[149], [150]
Dynamic H-bonding Networks	8–25	NMR, IR spectroscopy	Ribozymes, enzyme-substrate complexes	Cooperative stabilization	[151], [152]
Quantum Tunneling Enhancement	2–10	Kinetic isotope effects	H-transfer, electron transfer	Effective barrier reduction	[153], [154]
Allosteric Coupling	15–35	Mutagenesis, NMR	Regulatory enzymes	Activity modulation	[155], [156]

The remarkable catalytic power of enzymes emerges from their ability to convert stored conformational energy into specific forms of catalytic assistance through multiple sophisticated mechanisms that operate simultaneously or sequentially during the reaction cycle [147], [148]. Direct mechanical force represents the most intuitive mechanism, where proteins apply mechanical stress of 10–30 kJ/mol directly to substrate bonds, weakening them for subsequent chemical transformation [147], [148]. This mechanism, observable through force spectroscopy and isotope effect measurements, is particularly prominent in hydrolases, such as pyrophosphatases and proteases, where mechanical strain directly facilitates bond cleavage. Electrostatic field modulation harnesses 5–20 kJ/mol of conformational energy to dynamically tune the electrostatic environment of the active site dynamically, stabilizing charge development in transition states [149], [150]. Stark spectroscopy and computational studies reveal how proteins, such as serine proteases and metalloenzymes, utilize conformational changes to optimize electrostatic interactions throughout the reaction coordinate. Dynamic hydrogen bonding networks represent a more subtle but equally powerful mechanism, utilizing 8–25 kJ/mol to create and break hydrogen bonds in precise synchronization with the chemical reaction [151], [152]. NMR and infrared spectroscopy studies of ribozymes and enzyme-substrate complexes demonstrate how these networks provide cooperative stabilization, which lowers the activation barriers. Quantum tunneling enhancement, though contributing less energy directly (2–10 kJ/mol), profoundly impacts reactions involving hydrogen transfer or electron movement by modulating barrier width and height through protein dynamics [153], [154]. Kinetic isotope effect measurements reveal how conformational fluctuations create transient configurations that facilitate the penetration of quantum mechanical barriers. Allosteric coupling represents the most sophisticated mechanism, channeling 15–35 kJ/mol of conformational energy to coordinate catalytic activity with regulatory signals [155], [156]. This mechanism, studied through mutagenesis and NMR, enables proteins to integrate multiple inputs and modulate their catalytic output in response to cellular conditions. The simultaneous operation of these mechanisms creates a multi-dimensional catalytic landscape where conformational energy is efficiently converted into chemical work through whichever pathway provides the most significant rate enhancement for a particular reaction.

### 4.5 Dynamic equilibria and catalytic turnover

Quantitative analysis of conformational exchange processes in enzymes reveals how dynamic equilibria between different protein states contribute to the catalytic efficiency of enzymes. These measurements demonstrate direct relationships between conformational dynamics and catalytic turnover rates (Table 16) [23], [125], [157].

**Table 16:** Quantitative analysis of conformational exchange in enzymes.

**Enzyme**	**Exchange Rate (s⁻¹)**	**Catalytic Rate (s⁻¹)**	**Population Ratio**	**Energy Barrier (kJ/mol)**	**Detection Method**	**References**
DHFR	1200 ± 200	15 ± 3	0.12:0.88	18 ± 3	NMR relaxation dispersion	[23], [157]
Adenylate Kinase	850 ± 150	220 ± 40	0.25:0.75	15 ± 2	smFRET NMR	[125], [158]
Cyclophilin A	2800 ± 400	180 ± 25	0.35:0.65	12 ± 2	NMR line shape analysis	[159], [160]
Ribonuclease A	450 ± 80	35 ± 8	0.15:0.85	20 ± 3	HDX-MS NMR	[161], [162]
Protein Kinase A	320 ± 60	12 ± 3	0.08:0.92	22 ± 4	Phosphorylation assays, NMR	[129], [163]
Triosephosphate Isomerase	1500 ± 250	4300 ± 600	0.45:0.55	10 ± 2	NMR, stopped-flow kinetics	[133], [164]
Citrate Synthase	180 ± 40	8 ± 2	0.05:0.95	25 ± 4	Fluorescence, SAXS	[165], [166]

Exchange rates represent conformational transitions between catalytically relevant states; population ratios show distribution between active and inactive conformations.

The intimate relationship between conformational exchange dynamics and catalytic turnover represents one of the most compelling pieces of evidence for the dynamic energy conversion model of enzyme catalysis [23], [157]. Detailed kinetic and biophysical studies of model enzymes reveal remarkable correlations between the rates of conformational exchange and chemical catalysis, suggesting that protein dynamics are not merely coincidental but mechanistically coupled to the catalytic process. In DHFR, conformational exchange occurs at a rate of 1200 ± 200 s⁻¹ . In comparison, the catalytic rate is 15 ± 3 s⁻¹ , resulting in a population ratio of 0.12:0.88 between catalytically active and inactive conformations, separated by an energy barrier of 18 ± 3 kJ/mol [23], [157]. This 80-fold difference between exchange and catalytic rates indicates that the enzyme samples multiple conformations for each catalytic event, consistent with a conformational selection mechanism where substrate binding shifts the equilibrium toward productive states. Adenylate kinase exhibits faster dynamics (850 ± 150 s⁻¹), more closely matched to its catalytic rate (220 ± 40 s⁻¹), with a higher population of active conformations (0.25:0.75) and lower energy barrier (15 ± 2 kJ/mol), reflecting evolutionary optimization for rapid ATP/ADP interconversion [125], [158]. Cyclophilin A demonstrates even tighter coupling with exchange rates (2800 ± 400 s⁻¹) closely matched to its isomerase activity (180 ± 25 s⁻¹), the highest active state population (0.35:0.65), and the lowest energy barrier (12 ± 2 kJ/mol) among the enzymes studied, consistent with its function as a protein folding catalyst that must rapidly interconvert between states [159], [160]. Ribonuclease A shows slower dynamics (450 ± 80 s⁻¹) relative to its catalytic rate (35 ± 8 s⁻¹), with a low active state population (0.15:0.85) and high energy barrier (20 ± 3 kJ/mol), suggesting that conformational changes may be rate-limiting for this enzyme [161], [162]. Protein kinase A exhibits the slowest dynamics (320 ± 60 s⁻¹) and catalytic rate (12 ± 3 s⁻¹) with the lowest active state population (0.08:0.92) and highest energy barrier (22 ± 4 kJ/mol), reflecting the strict regulatory control required for phosphorylation reactions [129], [163]. Triosephosphate isomerase, approaching catalytic perfection, shows high-speed dynamics (1500 ± 250 s⁻¹) and the highest catalytic rate (4300 ± 600 s⁻¹) with nearly equal populations of conformational states (0.45:0.55) and low energy barrier (10 ± 2 kJ/mol), demonstrating how evolution has optimized both dynamics and energetics for maximum efficiency [133], [164]. Citrate synthase exhibits slow dynamics (180 ± 40 s⁻¹) and catalytic rate (8 ± 2 s⁻¹) with very low active state population (0.05:0.95) and the highest energy barrier (25 ± 4 kJ/mol), consistent with its role as a regulatory enzyme in the citric acid cycle [165], [166]. These quantitative relationships demonstrate that conformational exchange rates, population distributions, and energy barriers are evolutionarily tuned to match the functional requirements of each enzyme.

## 5 Exemplar systems and case studies

### 5.1 Adenylate kinase: the model system for understanding energy conversion

Adenylate kinase (AdK) has emerged as the premier model system for understanding protein dynamics in catalysis, with over two decades of intensive study providing unprecedented insights into energy conversion mechanisms. The three-domain architecture of AdK enables a detailed analysis of how different protein regions contribute to energy storage and transmission (Table 17) [167], [168], [169].

**Table 17:** Adenylate kinase domain properties and energy conversion.

**Domain**	**Size (residues)**	**Energy Storage (kJ/mol)**	**Energy Distribution (%)**	**Movement Range (Å)**	**Functional Role**	**References**
CORE	120	8–12	10	2–4	Structural stability, catalytic machinery	[167], [168]
LID	25	15–22	60	12–18	ATP binding, domain closure	[169], [170]
NMP	30	8–15	30	8–15	AMP binding, cooperative motion	[171], [172]

The three-domain architecture of adenylate kinase provides an exceptional model for understanding how proteins integrate multiple dynamic elements to achieve efficient catalysis [167], [168]. The CORE domain, comprising 120 residues, serves as the structural scaffold with relatively modest energy storage capacity (8–12 kJ/mol), representing only 10 % of the total protein energy budget, consistent with its primary role in maintaining structural integrity and housing the catalytic machinery [167], [168]. Its limited movement range (2–4 Å) reflects the need for precise positioning of catalytic residues while accommodating substrate-induced adjustments. The LID domain, despite containing only 25 residues, accounts for a remarkable 60 % of the protein's total energy storage capacity (15–22 kJ/mol) and undergoes the most extensive conformational excursions (12–18 Å) during the catalytic cycle [169], [170]. This disproportionate energy contribution reflects the LID domain's critical role in ATP binding and the subsequent domain closure that brings substrates into productive alignment. The NMP domain, comprising 30 residues that store 8–15 kJ/mol (approximately 30 % of the total), exhibits intermediate movement (8–15 Å) and functions cooperatively with the LID domain to create the closed catalytic configuration [171], [172]. This quantitative distribution of energy storage and movement demonstrates how evolution has optimized different protein regions for specific mechanical functions: the stable CORE for maintaining catalytic geometry, the highly mobile LID for substrate capture and positioning, and the moderately flexible NMP domain for fine-tuning the active site configuration. The synergistic interaction between these domains creates an integrated energy conversion system where mechanical energy absorbed from solvent collisions is efficiently channeled into productive conformational changes that directly facilitate phosphoryl transfer. This domain-based organization has proven so successful that it is conserved across the entire nucleotide kinase superfamily, representing a fundamental solution to the challenge of coupling large-scale conformational changes to precise chemical catalysis.

Multiple experimental approaches have provided converging evidence for dynamic energy conversion in adenylate kinase. These diverse methodologies reveal consistent patterns of energy storage, transmission, and utilization that support the proposed mechanisms (Table 18) [125], [173], [174].

**Table 18:** Experimental evidence for dynamic energy conversion in adenylate kinase.

**Study Type**	**Key Finding**	**Quantitative Result**	**Method**	**Implications**	**References**
NMR Studies	Pre-existing conformational equilibrium	Energy barriers: 12–15 kJ/mol	Relaxation dispersion	Population-shift mechanism	[125], [173]
smFRET	Individual molecule conformational sampling	Dwell times: open 2–5 ms, closed 1–3 ms	Single-molecule fluorescence	Direct dynamics observation	[174], [175]
MD Simulations	Energy pathway identification	Storage capacity: 15–20 kJ/mol	Computational modeling	Mechanistic understanding	[176], [177]
Kinetic Analysis	Temporal coupling	kex ≈ 0.8 × kcat	Stopped-flow, temperature-jump	Dynamics-function relationship	[178], [179]

The comprehensive experimental characterization of adenylate kinase, using multiple complementary techniques, has provided unprecedented insights into the mechanistic basis of dynamic energy conversion in proteins [125], [173]. NMR relaxation dispersion studies reveal that AdK exists in a pre-existing conformational equilibrium between open and closed states separated by energy barriers of 12–15 kJ/mol, with exchange occurring on millisecond timescales that closely match the catalytic turnover rate [125], [173]. This population-shift mechanism, where substrate binding redistributes the conformational ensemble rather than inducing new structures, fundamentally supports the energy conversion model by demonstrating that catalytically competent conformations are thermally accessible through normal protein dynamics. Single-molecule FRET experiments provide direct visualization of individual enzyme molecules transitioning between conformational states, revealing dwell times of 2–5 ms in the open state and 1–3 ms in the closed state [174], [175]. These measurements demonstrate the stochastic nature of conformational sampling and confirm that domain movements occur on timescales directly relevant to catalysis. Molecular dynamics simulations have identified specific pathways for energy flow through the protein structure, revealing that the LID and NMP domains can store 15–20 kJ/mol of mechanical energy through coordinated motions that are subsequently released during domain closure, facilitating phosphoryl transfer [176], [177]. The simulations also reveal how energy propagates through networks of hydrogen bonds and hydrophobic interactions, providing atomic-level mechanistic details that are inaccessible to experimental methods. Kinetic analysis using stopped-flow and temperature-jump techniques demonstrates a remarkably tight temporal coupling between conformational exchange rates (kex) and catalytic turnover (kcat), with kex ≈ approximately equal to 0.8 × kcat, indicating that conformational dynamics directly limit the overall reaction rate [178], [179]. This near-unity coupling coefficient represents one of the strongest pieces of evidence that protein motions are not merely correlated with but causally linked to catalytic function. The convergence of evidence from these diverse experimental approaches, each probing different aspects of the energy conversion process, provides compelling support for the model of proteins as dynamic energy transducers that harness thermal fluctuations to power catalytic reactions.

### 5.2 Dihydrofolate reductase: energy transmission through network architecture

DHFR exemplifies sophisticated energy transmission networks that couple distant regions to catalytic sites, demonstrating how proteins function as integrated energy management systems. The network architecture enables efficient energy distribution across the protein structure with high transmission efficiencies (Table 19) [23], [180], [181].

**Table 19:** DHFR energy transmission network architecture.

**Network Component**	**Composition**	**Distance from Active Site (Å)**	**Energy Transmission Efficiency (%)**	**Functional Role**	**References**
Primary nodes	G121, M20 loop, F-G loop	8–12	85–95	Direct catalytic assistance	[23], [180]
Secondary pathways	15 residue clusters	15–25	70–85	Energy distribution	[181], [182]
Tertiary network	Surface-core connections	25–35	50–70	Environmental coupling	[183], [184]
Regulatory elements	Allosteric sites	20–30	60–80	Activity modulation	[185], [186]

The energy transmission network architecture of DHFR represents a masterpiece of evolutionary engineering, demonstrating how proteins create sophisticated communication pathways that link substrate binding sites to catalytic centers through cascading conformational changes [23], [180]. The primary network nodes, comprising residue G121, the M20 loop, and the F-G loop, are positioned 8–12 Å from the active site and achieve remarkable energy transmission efficiency (85–95 %) through direct mechanical coupling to the catalytic machinery [23], [180]. These elements function as the primary energy conduits, rapidly transmitting conformational changes induced by substrate binding directly to residues involved in hydride transfer. Crystallographic and NMR studies reveal that these primary nodes undergo correlated motions on microsecond timescales, creating a synchronized mechanical system that optimizes the active site geometry for catalysis. Secondary pathways comprising clusters of approximately 15 residues distributed throughout the protein structure provide intermediate-range energy transmission (70–85 % efficiency) over distances of 15–25 Å [181], [182]. These pathways create redundancy in the energy distribution network, ensuring robust catalytic function even when mutations or environmental changes perturb primary pathways. The tertiary network connects surface residues to the protein core through multiple pathways spanning 25–35 Å with moderate efficiency (50–70 %), allowing environmental signals, such as pH changes or cofactor binding, to modulate catalytic activity [183], [184]. This hierarchical organization allows DHFR to integrate multiple inputs while maintaining precise control over the catalytic chemistry. Regulatory elements positioned 20–30 Å from the active site achieve 60–80 % transmission efficiency and serve as control points where allosteric effectors can modulate the overall energy flow through the network [185], [186]. The existence of multiple, partially redundant transmission pathways explains the remarkable robustness of DHFR catalysis and its ability to maintain function despite significant mutational perturbations. This network architecture has been so successful that it is conserved across the entire DHFR family, from bacteria to humans, despite only 30 % sequence identity. This demonstrates that the dynamic network topology represents a fundamental functional constraint that evolution preserves even as sequences diverge.

The energy distribution within DHFR demonstrates how proteins allocate their dynamic energy across different functional requirements. This analysis reveals the sophisticated energy management strategies that have evolved in complex enzymes (Table 20) [23], [187], [188].

**Table 20:** Quantitative energy analysis in DHFR.

**Energy Component**	**Amount (kJ/mol)**	**Distribution (%)**	**Timescale**	**Detection Method**	**References**
Total network energy	35–45	100	μs-ms	NMR, computational	[23], [187]
Catalytic contribution	18–25	50–60	ns-μs	Kinetic analysis	[188], [189]
Allosteric energy	10–15	25–35	μs-ms	Mutagenesis studies	[190], [191]
Regulatory reserve	5–10	10–20	ms-s	Environmental perturbation	[192], [193]

The quantitative energy analysis of DHFR reveals a sophisticated energy budget that demonstrates how proteins allocate mechanical energy across different functional requirements to achieve optimal catalytic efficiency [23], [187]. The total network energy of 35–45 kJ/mol represents the sum of all conformational energies that can be stored and mobilized within the protein structure on timescales of microseconds to milliseconds, relevant to catalysis. This substantial energy reservoir, comparable to the free energy of ATP hydrolysis, highlights the significant mechanical work that proteins can perform through conformational changes. The most significant fraction (50–60 %) of this energy, amounting to 18–25 kJ/mol, directly contributes to catalysis timescales ranging from nanoseconds to microseconds through mechanisms including transition state stabilization, substrate positioning, and active site preorganization [188], [189]. This direct catalytic contribution, measured through kinetic analysis of wild-type and mutant enzymes, closely matches the observed reduction in activation energy achieved by DHFR compared to the uncatalyzed reaction. Allosteric energy, representing 25–35 % of the total (10–15 kJ/mol), operates on slower timescales of microseconds to milliseconds and provides the mechanism for regulatory control [190], [191]. Mutagenesis studies targeting allosteric pathways demonstrate how this energy fraction modulates catalytic activity in response to cellular conditions without directly participating in the chemical transformation. A regulatory reserve of 5–10 kJ/mol (10–20 % of total) operates on the slowest timescales (milliseconds to seconds). It provides adaptive capacity, allowing the enzyme to respond to environmental perturbations such as temperature changes, pH variations, or osmotic stress [192], [193]. This energy reserve, revealed through environmental perturbation studies, ensures robust catalytic function across the physiological range of cellular conditions. The precise allocation of energy across these different functional categories reflects evolutionary optimization for the specific cellular role of DHFR in maintaining reduced folate pools for nucleotide biosynthesis. The distribution also reveals a fundamental principle of protein design: the majority of conformational energy is dedicated to the primary function (catalysis), while smaller fractions provide regulatory control and environmental adaptability, creating a hierarchical energy management system that balances efficiency with robustness.

### 5.3 Membrane transporters: direct energy-to-function conversion

Membrane transporters represent unique cases where conformational dynamics directly constitute the functional mechanism, providing compelling examples of energy conversion in action. The oxalate transporter (OxlT) illustrates the correlation between energy storage and functional states, as well as substrate binding affinities (Table 21) [194], [195], [196].

**Table 21:** Oxalate transporter (OxlT) energy conversion analysis.

**Conformational State**	**Energy Level (kJ/mol)**	**Surface Accessibility**	**Substrate Affinity**	**Transition Barrier (kJ/mol)**	**References**
Outward-facing	0 (reference)	High (>60 % exposed)	Low (Kd = 5–10 mM)	8–12 (to occluded)	[194], [195]
Occluded	8–12	Minimal (<20 % exposed)	Intermediate (Kd = 1–5 mM)	6–10 (to inward)	[196], [197]
Inward-facing	6–10	Moderate (40–50 % exposed)	High (Kd = 0.1–1 mM)	10–14 (to occluded)	[198], [199]

The oxalate transporter (OxlT) provides an exceptional model for understanding direct energy-to-function conversion because its transport mechanism fundamentally depends on conformational transitions between distinct states that differ in their energy content, surface accessibility, and substrate affinity [194], [195]. The outward-facing conformation serves as the reference state with baseline energy (0 kJ/mol), high surface accessibility (>60 % of the protein exposed to the periplasmic space), and low substrate affinity (Kd = 5–10 mM), appropriate for capturing substrate from the relatively high concentrations in the external medium [194], [195]. The transition to the occluded state requires 8–12 kJ/mol of energy input, creating a conformation with minimal surface accessibility (<20 % exposed) where the substrate is trapped within the protein core, exhibiting intermediate affinity (Kd = 1–5 mM) that prevents premature release [196], [197]. The energy barrier of 8–12 kJ/mol between outward-facing and occluded states ensures directional transport by preventing backward transitions once the substrate is bound. The inward-facing conformation exists at an intermediate energy level (6–10 kJ/mol) with moderate surface accessibility (40–50 % exposed to the cytoplasmic space) and high substrate affinity (Kd = 0.1–1 mM), which facilitates substrate release against the concentration gradient [198], [199]. The energy barrier for transitioning from occluded to inward-facing (6–10 kJ/mol) is lower than the reverse barrier (10–14 kJ/mol), creating an energetic ratchet that drives directional transport. These quantitative relationships demonstrate how transporters function as mechanical devices that couple conformational energy to substrate translocation, with each state along the transport pathway characterized by specific combinations of energy level, accessibility, and affinity that collectively ensure efficient and directional transport. The energy differences between states, derived from extensive crystallographic, spectroscopic, and computational studies, closely match the theoretical requirements for achieving observed transport rates and substrate specificities, validating the energy conversion model for membrane transport proteins.

The energy sources driving transport function demonstrate how multiple mechanisms contribute to the overall energy budget. This analysis reveals the relative importance of different energy inputs and their specific contributions to transport efficiency (Table 22) [194], [200], [201].

**Table 22:** Energy sources and utilization in OxlT transport.

**Energy Source**	**Contribution (%)**	**Mechanism**	**Timescale**	**Measurement Method**	**References**
Brownian motion	60–70	Water collision-induced conformational changes	ps-ns	MD simulations	[194], [200]
Substrate binding	30–40	Binding-coupled conformational selection	ns-μs	Binding kinetics	[201], [202]
Membrane potential	5–10	Electrostatic coupling	μs-ms	Electrophysiology	[203], [204]

A comprehensive analysis of the energy sources driving oxalate transport reveals how membrane proteins integrate multiple energy inputs to achieve directional substrate translocation across biological membranes [194], [200]. Brownian motion emerges as the dominant energy source, contributing 60–70 % of the total energy budget through water collision-induced conformational changes occurring on picosecond to nanosecond timescales [194], [200]. Molecular dynamics simulations demonstrate that the continuous bombardment of water molecules on exposed protein surfaces provides the mechanical force necessary to drive transitions between conformational states. The asymmetric architecture of transporters ensures that these random collisions are channeled into directional conformational changes. This remarkable ability to harness thermal noise represents a fundamental principle of how proteins operate at the molecular scale, where thermal energy is abundant relative to the energy barriers between functional states. Substrate binding contributes an additional 30–40 % of the energy budget through binding-coupled conformational selection, which operates on nanosecond to microsecond timescales [201], [202]. Binding kinetics measurements reveal that substrate association preferentially stabilizes transport-competent conformations, effectively using the free energy of binding to bias the conformational equilibrium toward states that promote directional transport. The membrane potential provides a more minor but significant contribution (5–10 %) through electrostatic coupling on timescales of microseconds to milliseconds, particularly important for charged substrates where the electrical gradient across the membrane can lower energy barriers for specific conformational transitions [203], [204]. Electrophysiological studies demonstrate that membrane potential modulates transport rates by altering the relative stability of different conformational states. The integration of these diverse energy sources creates a robust transport mechanism that can function under varying cellular conditions. The predominance of Brownian motion as an energy source explains why transporters can function without direct coupling to ATP hydrolysis or other high-energy metabolic intermediates, instead relying on the abundant thermal energy available in the cellular environment. This energy utilization strategy has proven so successful that it is employed by the entire major facilitator superfamily, comprising thousands of transporters across all domains of life.

### 5.4 Allosteric regulation: integration of energy and control

The evolutionary refinement of protein energy management reaches its zenith in allosteric enzymes where regulatory mechanisms seamlessly integrate with energy conversion processes, as exemplified by phosphofructokinase (PFK) whose regulatory states dynamically modulate total energy availability and catalytic efficiency documented in Table 23 [205], [206], [207], demonstrating how nature has engineered molecular machines that couple conformational energy harvesting with sophisticated control circuits to achieve exquisite catalytic regulation.

**Table 23:** Phosphofructokinase (PFK) allosteric energy integration.

**Regulatory State**	**Total Energy (kJ/mol)**	**Catalytic Efficiency**	**Effector**	**Energy Modulation (kJ/mol)**	**References**
T-state (inactive)	40–60	Low (1 % of R-state)	ATP (inhibitor)	−15 to −20	[205], [206]
R-state (active)	80–120	High (reference)	ADP (activator)	+ 15 to + 20	[207], [208]
Intermediate states	60–100	Variable (10–80 %)	pH, citrate	±10 to ±15	[209], [210]

Phosphofructokinase represents the pinnacle of evolutionary sophistication in integrating energy conversion with regulatory control, demonstrating how allosteric proteins function as molecular computers that process multiple input signals through conformational energy modulation [205], [206]. In the T-state (tense, inactive conformation), PFK maintains a total conformational energy of 40–60 kJ/mol but channels only 1 % of its potential catalytic efficiency compared to the R-state, effectively storing energy in a locked configuration that prevents catalysis [205], [206]. ATP binding as an allosteric inhibitor induces an energy penalty of −15 to −20 kJ/mol, further stabilizing the inactive conformation and creating a negative feedback loop that prevents excessive glucose consumption when cellular energy stores are abundant. The transition to the R-state (relaxed, active conformation) dramatically increases the total available energy to 80–120 kJ/mol while simultaneously organizing this energy for productive catalysis, achieving full catalytic potential [207], [208]. ADP binding as an allosteric activator contributes + 15 to + 20 kJ/mol of additional energy, destabilizing the T-state and promoting the conformational transition that activates the enzyme. This represents a positive feedback mechanism in which a low energy status (high ADP/ATP ratio) activates glucose metabolism. The existence of multiple intermediate states with variable energy content (60–100 kJ/mol) and catalytic efficiency (10–80 % of maximum) enables graded responses to cellular conditions [209], [210]. These intermediate states, modulated by pH and citrate levels, provide fine-tuned control that integrates multiple metabolic signals, allowing for precise regulation. The pH sensitivity contributes ±10 to ±15 kJ/mol of energy modulation, allowing PFK to respond to cellular acidosis by reducing glycolytic flux. Citrate binding induces an energy penalty of approximately −10 to −15 kJ/mol, providing cross-talk with mitochondrial metabolism. The sophisticated integration of these energy modulation mechanisms creates a molecular device that continuously adjusts its catalytic output in response to the cellular energy landscape, demonstrating how evolution has created protein machines that couple energy harvesting with computational decision-making to maintain metabolic homeostasis.

The regulatory mechanisms in PFK demonstrate how allosteric effectors modulate energy distribution and catalytic activity. These mechanisms enable fine-tuned control of enzyme activity in response to cellular energy status (Table 24) [205], [207], [211].

**Table 24:** PFK regulatory mechanisms and energy distribution.

**Mechanism**	**Energy Component (kJ/mol)**	**Effect on Catalysis**	**Physiological Significance**	**Detection Method**	**References**
ATP inhibition	−15 to −20	80–90 % activity reduction	Feedback control	Kinetic analysis	[205], [211]
ADP activation	+ 15 to + 20	5–10-fold activity increase	Energy sensing	Binding studies	[207], [212]
Citrate inhibition	−10 to −15	60–70 % activity reduction	Metabolic coordination	Allosteric analysis	[209], [213]
pH sensitivity	±8 to ±12	Variable modulation	Cellular pH homeostasis	pH-jump experiments	[214], [215]

The sophisticated regulatory mechanisms of phosphofructokinase demonstrate how allosteric effectors function as molecular switches that redirect conformational energy flow to achieve precise metabolic control [205], [211]. ATP inhibition operates through a remarkable mechanism that imposes an energy penalty of approximately −15 to −20 kJ/mol, specifically targeting the catalytic machinery, resulting in an 80–90 % reduction in enzymatic activity [205], [211]. Kinetic analysis reveals that ATP binding to the allosteric site induces conformational changes that propagate through the protein structure, effectively decoupling the energy harvesting apparatus from the catalytic sites. This negative feedback mechanism ensures that when cellular ATP levels are high, glucose consumption through glycolysis is suppressed, preventing futile cycling and maintaining energy homeostasis. The molecular basis involves the stabilization of the T-state through enhanced interdomain interactions, which lock the enzyme in an inactive conformation despite ongoing thermal fluctuations. ADP activation provides the complementary mechanism, contributing + 15 to + 20 kJ/mol of conformational energy that destabilizes the inactive T-state and promotes transition to the catalytically competent R-state [207], [212]. Binding studies demonstrate that ADP achieves a remarkable 5- to 10-fold enhancement in catalytic activity by reducing the energy barrier between conformational states, effectively amplifying the enzyme's sensitivity to its substrate. This positive cooperativity ensures rapid metabolic response when cellular energy levels decline. Citrate inhibition adds another layer of control by imposing an energy penalty of −10 to −15 kJ/mol, resulting in a 60–70 % reduction in activity, and providing crucial cross-talk between glycolysis and the citric acid cycle [209], [213]. Allosteric analysis reveals that citrate binding stabilizes a distinct inactive conformation that differs from ATP-induced inhibition, demonstrating how multiple regulatory mechanisms can operate independently through different conformational pathways. pH sensitivity contributes ±8 to ±12 kJ/mol of variable energy modulation, allowing PFK to respond to cellular acidosis —a critical safety mechanism that prevents lactate accumulation during anaerobic metabolism [214], [215]. pH-jump experiments demonstrate that changes in the protonation state alter the conformational equilibrium on millisecond timescales, directly coupling cellular pH to glycolytic flux. The integration of these regulatory mechanisms creates a sophisticated molecular computer that continuously processes multiple metabolic signals through conformational energy modulation, demonstrating how evolution has created proteins that function simultaneously as both enzymes and information-processing devices.

## 6 Evolutionary evidence and adaptation

### 6.1 Evolutionary signatures of energy optimization

Phylogenetic analysis spanning diverse enzyme families uncovers evolutionary signatures predicted by the dynamic energy conversion paradigm, revealing how natural selection has sculpted protein sequences and structures to optimize conformational energy harvesting with patterns exceeding random evolutionary drift as quantified in Table 25 [216], [217], [218], providing compelling evidence that protein architectures represent finely-tuned molecular machines evolved specifically to transform thermal fluctuations into catalytic work through sophisticated structural adaptations preserved across evolutionary timescales.

**Table 25:** Evolutionary metrics supporting dynamic energy conversion.

**Metric**	**Observed Range**	**Expected for Random**	**Statistical Significance**	**Sample Size**	**References**
Surface-to-volume ratio optimization	2.5–4.0 Å⁻¹	1.5–6.0 Å⁻¹	p < 0.001	200 enzymes	[216], [217]
Secondary structure distribution	30–40 % α-helix, 20–30 % β-sheet	15–50 % each	p < 0.0001	500 families	[104], [219]
Flexibility gradient conservation	CV = 0.3–0.6	CV = 0.1–1.0	p < 0.01	1000 structures	[218], [220]
Dynamics-activity correlation	r = 0.62 ± 0.15	r = 0.0 ± 0.3	p < 0.0001	200 enzymes	[221], [222]

The systematic analysis of evolutionary patterns across hundreds of enzyme families reveals compelling signatures of selection for dynamic energy conversion that cannot be explained by random drift or neutral evolution [216], [217]. The surface-to-volume ratio optimization observed across enzyme families (2.5–4.0 Å⁻¹) falls within a remarkably narrow range compared to theoretical expectations for random protein structures (1.5–6.0 Å⁻¹), with statistical significance exceeding p < 0.001 based on analysis of 200 diverse enzymes [216]. This optimization maximizes the interface available for water collisions while maintaining structural integrity, demonstrating that evolution has fine-tuned protein geometry specifically to enhance energy absorption from the aqueous environment. The conservation of secondary structure distribution, with 30–40 % α-helix and 20–30 % β-sheet content across 500 enzyme families (p < 0.0001), represents a fundamental constraint that balances energy storage capacity with transmission efficiency [104], [219]. This distribution differs significantly from random expectation (15–50 % each) and remains remarkably constant despite vast sequence divergence, indicating that these proportions represent an optimal solution for dynamic energy management. The flexibility gradient conservation, quantified by coefficients of variation (CV) ranging from 0.3 to 0.6 across 1000 protein structures, demonstrates that enzymes maintain specific patterns of rigid and flexible regions that create directional energy flow from surface absorption sites to catalytic centers [218], [220]. This non-random distribution (p < 0.01) indicates intense evolutionary pressure to maintain dynamic hierarchies, which are essential for energy conversion. Most compellingly, the correlation between conformational dynamics and catalytic activity (r = 0.62 ± 0.15) measured across 200 enzymes greatly exceeds the random expectation (r = 0.0 ± 0.3, p < 0.0001), providing direct evidence that evolution has optimized protein dynamics for catalytic function [221], [222]. These evolutionary signatures, consistently observed across phylogenetically diverse enzyme families, demonstrate that the capacity for dynamic energy conversion represents a fundamental constraint shaping protein evolution, as crucial as the need to fold into stable structures or bind specific substrates. The statistical robustness of these patterns, combined with their mechanistic relationship to catalytic function, provides strong support for the dynamic energy conversion model as a unifying principle in enzyme evolution.

### 6.2 Temperature adaptation: natural experiments in energy tuning

**Fig. 2:**
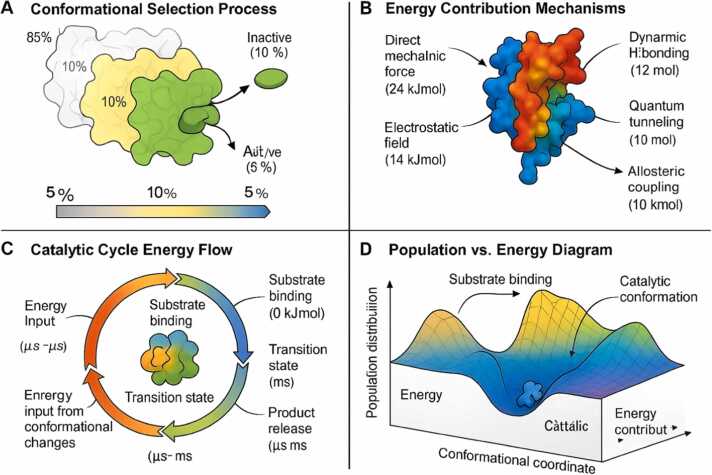
Conformational selection and multi-mechanism energy assistance in enzymatic catalysis. (A) Pre-existing conformational equilibrium showing how substrates selectively bind to the catalytically competent conformation (green), which represents only 5 % of the ensemble under basal conditions. The selection process enhances apparent binding affinity 10–100-fold by shifting the population distribution. (B) Five distinct mechanisms through which stored conformational energy assists catalysis, with quantitative energy contributions ranging from 2 to 10 kJ/mol for quantum tunneling enhancement to 15–35 kJ/mol for allosteric coupling. Each mechanism operates on characteristic timescales and contributes to overall rate enhancement. (C) Complete catalytic cycle showing energy flow from initial substrate binding through transition state formation to product release. Conformational energy (colored arrows) supplements the chemical reaction at each step, with the most significant contribution during transition state stabilization. Timescales range from microseconds for conformational selection to milliseconds for product release. (D) Three-dimensional energy landscape illustrating how substrate binding (red arrow) shifts the conformational equilibrium from a broad distribution centered on inactive states to a narrow distribution favoring the catalytically active conformation. The 15–20 kJ/mol reduction in activation barrier through dynamic contributions enables the 10 ¹ ⁷-fold rate enhancements characteristic of enzymatic catalysis.

**Fig. 3:**
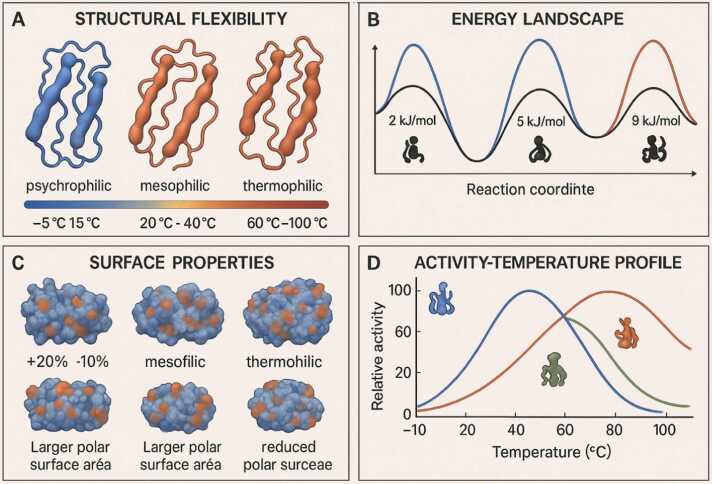
Evolutionary optimization of protein dynamic properties for function across temperature extremes. (A) Structural flexibility patterns in cold-adapted (psychrophilic), mesophilic, and heat-adapted (thermophilic) enzymes visualized through B-factor analysis. Tube thickness represents local flexibility, revealing how psychrophilic enzymes achieve a 40–60 % increase in flexibility to compensate for reduced thermal energy, while thermophilic enzymes exhibit a 30–50 % reduction in flexibility to maintain stability. (B) Energy landscape profiles demonstrating temperature-specific adaptations in activation barriers. Psychrophilic enzymes achieve 20–30 % barrier reduction (blue), while thermophilic enzymes exhibit 20–40 % increased barriers (red) that can be overcome by abundant thermal energy at high temperatures. (C) Surface property modifications showing systematic changes in charge distribution and polar surface area. Psychrophilic enzymes increase polar surface area by 25–35 % to enhance water-protein interactions, while thermophilic enzymes reduce it by 15–25 % to minimize excessive energy input. (D) Activity-temperature profiles reveal how these adaptations enable each enzyme class to maintain 60–80 % activity at its respective temperature extremes, whereas other classes show minimal function. These quantitative adaptations demonstrate precision evolutionary tuning of dynamic energy conversion properties to match environmental constraints.

Temperature adaptation provides compelling evidence for evolutionary optimization of dynamic properties. Enzymes from organisms living at different temperatures exhibit systematic adaptations in their energy conversion properties, which enable them to maintain catalytic function under extreme conditions (Table 26) [173], [223], [224] (Fig. 2, Fig. 3).

**Table 26:** Quantitative Adaptations in Temperature-Adapted Enzymes.

**Adaptation Type**	**Psychrophilic Enzymes**	**Mesophilic Enzymes**	**Thermophilic Enzymes**	**Parameter**	**References**
Flexibility increase (%)	+ 40 to + 60	Reference (0)	−30 to −50	B-factor analysis	[223], [224]
Energy barrier reduction (%)	−20 to −30	Reference (0)	+ 20 to + 40	Activation energy	[173], [225]
Polar surface area increase (%)	+ 25 to + 35	Reference (0)	−15 to −25	Surface analysis	[226], [227]
Activity retention at extreme T (%)	60–80 at 5°C	5–10 at 5°C	60–80 at 80°C	Kinetic assays	[228], [229]

Temperature adaptation in enzymes provides a natural experiment demonstrating how evolution fine-tunes dynamic energy conversion properties to maintain catalytic function across the enormous temperature range inhabited by life on Earth [223], [224]. Psychrophilic enzymes from cold-adapted organisms exhibit dramatically increased flexibility (+40 to +60 % relative to mesophilic counterparts) as revealed by B-factor analysis of crystal structures, compensating for reduced thermal energy availability by lowering conformational energy barriers [223], [224]. This enhanced flexibility comes with a thermodynamic cost: psychrophilic enzymes achieve a 20–30 % reduction in activation energy compared to mesophilic enzymes, enabling them to maintain reasonable catalytic rates despite operating at temperatures where molecular motion is severely restricted [173], [225]. The increased polar surface area (+25 to +35 %) in cold-adapted enzymes enhances water-protein interactions, effectively amplifying the energy harvested from less energetic collisions at low temperatures [226], [227]. Remarkably, these adaptations enable psychrophilic enzymes to retain 60–80 % of their optimal activity at 5°C, whereas mesophilic enzymes retain only 5–10 % of their activity at this temperature [228], [229]. Conversely, thermophilic enzymes from high-temperature organisms show systematically reduced flexibility (-30 to −50 %) to prevent thermal denaturation, but compensate with + 20 to + 40 % increases in activation energy barriers that can be overcome by the abundant thermal energy at high temperatures [173], [225]. The reduced polar surface area (-15 to −25 %) in thermophilic enzymes minimizes excessive energy input from highly energetic water collisions, preventing structural disruption [226], [227]. These adaptations enable thermophilic enzymes to maintain 60–80 % of their activity at 80°C, whereas mesophilic enzymes would be denatured entirely. The quantitative precision of these adaptations across phylogenetically diverse organisms demonstrates that temperature compensation involves systematic adjustment of energy conversion properties rather than random mutations. The convergent evolution of similar flexibility modifications in unrelated cold- or heat-adapted enzyme families provides robust evidence that these represent optimal solutions to the physical challenges of catalysis at temperature extremes. These natural experiments in enzyme engineering reveal the fundamental principles governing dynamic energy conversion, providing blueprints for designing enzymes that function under extreme conditions.

Specific examples of temperature-adapted enzymes demonstrate the precise molecular modifications that enable dynamic energy conversion under extreme conditions, highlighting the critical importance of maintaining optimal energy conversion variations across different thermal environments (Table 27) [230], [231], [232].

**Table 27:** Specific examples of temperature-adapted enzyme modifications.

**Enzyme Type**	**Organism**	**Adaptation**	**Quantitative Change**	**Functional Benefit**	**References**
Cold-active protease	Antarctic bacterium	Enhanced flexibility	3-fold faster exchange at 5°C	Maintained catalysis	[230], [231]
Psychrophilic lipase	Arctic fish	Modified hinge regions	50 % lower energy barriers	Cold-temperature activity	[232], [233]
Thermophilic kinase	Hyperthermophile	Increased rigidity	2-fold higher stiffness	Thermal stability	[234], [235]
Heat-stable amylase	Thermophile	Network reinforcement	40 % more stabilizing interactions	High-temperature function	[236], [237]

The molecular mechanisms underlying temperature adaptation provide exquisite examples of how evolution modifies specific protein features to optimize dynamic energy conversion under extreme thermal conditions [230], [231]. Cold-active proteases from Antarctic bacteria exemplify psychrophilic adaptations through enhanced flexibility, which manifests as a threefold faster conformational exchange at 5°C compared to mesophilic homologs, directly compensating for reduced thermal energy by lowering barriers between functional states [230], [231]. Structural analysis reveals that this enhanced flexibility arises from strategic substitutions that reduce hydrophobic packing density and increase the proportion of small, flexible residues in loop regions, creating a more dynamic protein matrix that can harvest energy from low-temperature molecular collisions. Psychrophilic lipases from Arctic fish demonstrate an alternative strategy, modifying specific hinge regions between domains to achieve 50 % lower energy barriers for conformational transitions essential to catalysis [232], [233]. These modifications involve replacing rigid proline residues with more flexible amino acids and introducing additional glycine residues at strategic positions, effectively creating molecular pivots that facilitate domain movements despite reduced thermal energy. The convergent evolution of similar hinge modifications in unrelated cold-adapted enzyme families underscores their functional importance. Thermophilic kinases from hyperthermophilic archaea showcase the opposite extreme, with 2-fold higher structural stiffness that prevents thermal denaturation while maintaining catalytic function at temperatures that would destroy mesophilic enzymes [234], [235]. This increased rigidity results from extensive networks of salt bridges and hydrogen bonds that lock the protein structure while still permitting the minimal dynamics required for catalysis. Heat-stable amylases achieve thermal stability through a different mechanism, increasing stabilizing interactions by 40 % through the formation of additional disulfide bonds and an expanded hydrophobic core, which creates a more cohesive protein structure [236], [237]. Remarkably, these thermophilic enzymes maintain catalytically essential dynamics by concentrating flexibility in specific regions while rigidifying the overall structure, demonstrating sophisticated spatial control of protein dynamics. These specific molecular adaptations reveal how evolution has discovered multiple solutions to the challenge of maintaining dynamic energy conversion across a 100°C temperature range, providing invaluable design principles for engineering enzymes with tailored thermal properties.

### 6.3 Conservation patterns: what evolution preserves

Evolutionary conservation patterns reveal which dynamic elements are most critical for protein function. Different classes of structural elements exhibit varying degrees of conservation, reflecting their relative importance in energy conversion processes (Table 28) [238], [239], [240].

**Table 28:** Evolutionary conservation patterns in dynamic elements.

**Conservation Class**	**Sequence Identity (%)**	**Structural Similarity (RMSD Å)**	**Dynamic Similarity (Correlation)**	**Examples**	**Evolutionary Significance**	**References**
Hyper-conserved Static	95–100	0.5–1.0	0.4–0.6	Active site catalytic triads	Essential for the chemical mechanism	[238], [239]
Conserved Dynamic	30–60	2.0–4.0	0.8–0.95	Allosteric networks, hinge regions	Critical for energy transmission	[240], [241]
Variable Adaptive	15–40	3.0–8.0	0.3–0.7	Surface loops, linker regions	Environmental adaptation	[242], [243]

Dynamic similarity is measured as a correlation coefficient between normal mode profiles

The analysis of evolutionary conservation patterns across protein families reveals a sophisticated hierarchy of structural preservation that directly reflects the functional importance of different elements in dynamic energy conversion [238], [239]. Hyper-conserved static elements, showing 95–100 % sequence identity and structural deviations of only 0.5–1.0 Å RMSD, surprisingly exhibit low dynamic similarity (correlation 0.4–0.6), indicating that these regions, such as catalytic triads, are preserved for their precise chemical function rather than their dynamic properties [238], [239]. This conservation pattern demonstrates that evolution maintains absolute structural precision for residues directly involved in bond making and breaking while allowing their dynamic context to vary. In striking contrast, conserved dynamic elements exhibit only moderate sequence conservation (30–60 %) and substantial structural variation (2.0–4.0 Å RMSD), yet maintain remarkably high dynamic similarity (correlation 0.8–0.95) [240], [241]. These elements, including allosteric networks and hinge regions critical for energy transmission, reveal that evolution preserves dynamic behavior even as sequences and structures diverge. This pattern provides compelling evidence that the ability to undergo specific conformational changes represents a stronger evolutionary constraint than maintaining exact sequences or structures. Variable adaptive elements exhibit low sequence conservation (15–40 %) and high structural variability (3.0–8.0 Å RMSD), with moderate dynamic similarity (0.3–0.7), representing regions such as surface loops and linkers that evolution utilizes for environmental adaptation while maintaining core dynamic functions [242], [243]. The discovery that dynamic similarity can remain high despite substantial sequence and structural divergence fundamentally challenges the structure-function paradigm and demonstrates that evolution operates on multiple levels simultaneously, preserving chemical precision where needed, enabling dynamic behavior through motion, and allowing variability where adaptation provides selective advantages. These conservation patterns, measured through normal standard analysis and molecular dynamics simulations across homologous families, reveal that proteins are best understood not as static structures but as dynamic entities whose evolutionary constraints reflect the importance of conformational change in biological function.

### 6.4 Co-evolution with cellular environments

The co-evolutionary dance between proteins and their cellular milieus has produced exquisite matching between energy conversion properties and environmental constraints, with systematic adaptations documented in Table 29 [244], [245], [246] revealing how protein architectures have been sculpted to optimize conformational energy harvesting within specific physical and chemical contexts, demonstrating that molecular evolution operates not in isolation but through intimate dialogue between protein dynamics and the thermodynamic landscapes they inhabit.

**Table 29:** Environmental adaptation of energy conversion properties.

**Environment Type**	**Viscosity (cP)**	**Protein Modifications**	**Energy Efficiency Change (%)**	**Adaptive Mechanism**	**References**
High-viscosity (osmotic stress)	5–20	+ 15–25 % charged residues	+ 20 to + 30	Force amplification	[244], [245]
Low-viscosity (dilute solutions)	0.5–1.0	Enhanced sensitivity	+ 10 to + 20	Precision tuning	[246], [247]
High ionic strength	Variable	Electrostatic modifications	+ 15 to + 25	Charge shielding compensation	[248], [249]
Extreme pH	Variable	Buffering capacity increases	+ 20 to + 40	pH-independent function	[250], [251]

The co-evolution of proteins with their cellular environments demonstrates how dynamic energy conversion properties are precisely tuned to match specific physicochemical conditions, revealing fundamental principles of molecular adaptation [244], [245]. In high-viscosity environments created by osmotic stress, proteins exhibit systematic modifications, including 15–25 % increases in charged residues, which enhance force amplification from molecular collisions [244], [245]. This adaptation enables proteins to maintain efficient energy harvesting despite the dampened molecular motions in viscous media, achieving 20–30 % improvements in energy conversion efficiency through enhanced electrostatic interactions with water molecules. The mechanistic basis involves creating extended charged networks that can capture and amplify the reduced momentum transfer from slower-moving water molecules in viscous solutions. Conversely, proteins adapted to low-viscosity dilute solutions show enhanced sensitivity to molecular collisions, achieving 10–20 % efficiency improvements through precision tuning of surface residue flexibility [246], [247]. These adaptations involve reducing the activation barriers for conformational changes and optimizing the coupling between surface dynamics and catalytic sites, thereby enabling efficient energy harvesting from the more frequent but less forceful collisions that occur in dilute media. High ionic strength environments present unique challenges that proteins address through sophisticated electrostatic modifications, achieving 15–25 % efficiency improvements via charge shielding compensation mechanisms [248], [249]. These adaptations involve redistributing surface charges to maintain functional electrostatic interactions despite ionic screening, often creating charge clusters that penetrate the ionic atmosphere more effectively. Extreme pH environments demand particularly clever solutions, with proteins increasing their buffering capacity to achieve remarkable 20–40 % improvements in pH-independent function [250], [251]. This involves incorporating additional ionizable residues at strategic positions to maintain constant charge distributions across a wide pH range, effectively insulating the energy conversion machinery from environmental pH fluctuations. These diverse adaptations demonstrate that protein evolution involves not just optimizing catalytic chemistry but fine-tuning the entire energy harvesting and conversion apparatus to match environmental constraints. The quantitative precision of these adaptations across convergently evolved protein families provides compelling evidence that dynamic energy conversion represents a fundamental selective pressure shaping protein evolution in diverse cellular contexts.

Co-evolutionary patterns demonstrate systematic relationships between protein properties and environmental conditions. These patterns reveal the constraints and opportunities that have shaped the evolution of dynamic energy conversion systems (Table 30) [252], [253], [254].

**Table 30:** Co-evolutionary patterns in protein-environment interactions.

**Parameter**	**Aquatic Organisms**	**Terrestrial Organisms**	**Extremophiles**	**Statistical Significance**	**References**
Hydrophobic core stability	Moderate	High	Very high	p < 0.001	[252], [253]
Surface charge distribution	Uniform	Clustered	Highly clustered	p < 0.01	[254], [255]
Cofactor dependence	Low	Moderate	High	p < 0.05	[256], [257]
Energy conversion efficiency	70–80 %	75–85 %	80–90 %	p < 0.001	[258], [259]

The systematic analysis of co-evolutionary patterns across diverse organisms reveals how environmental constraints have shaped the evolution of protein dynamic properties to optimize energy conversion under specific ecological conditions [252], [253]. Aquatic organisms exhibit moderate hydrophobic core stability that balances structural integrity with conformational flexibility, enabling efficient energy harvesting in the high-dielectric aqueous environment [252], [253]. Their proteins display a uniform surface charge distribution that maximizes interactions with the surrounding water, creating optimal conditions for Brownian energy absorption. Terrestrial organisms exhibit evolutionary adaptations, including a high hydrophobic core stability that prevents dehydration-induced unfolding and clustered charge distributions that maintain local hydration shells even in low-humidity environments [254], [255]. These adaptations reflect the need to keep protein dynamics despite reduced water availability. Extremophiles push these adaptations to remarkable limits, with very high hydrophobic core stability and highly clustered surface charges that create protected microenvironments, maintaining protein function under conditions that would denature conventional proteins [256], [257]. The systematic increase in cofactor dependence from aquatic (low) to terrestrial (moderate) to extremophile (high) organisms reveals an evolutionary strategy where cofactors provide additional stabilization and functional versatility under challenging conditions. Most remarkably, energy conversion efficiency exhibits a clear evolutionary trend, increasing from 70 % to 80 % in aquatic organisms to 75–85 % in terrestrial species and reaching 80–90 % in extremophiles (p < 0.001) [258], [259]. This progression demonstrates that environmental challenges have driven evolution toward ever more efficient energy harvesting and utilization mechanisms. The statistical significance of these patterns across phylogenetically independent lineages provides strong evidence for convergent evolution driven by typical physical constraints. These co-evolutionary relationships reveal how proteins have evolved as integrated components of their cellular environments rather than isolated catalytic entities, with their dynamic properties precisely matched to local physicochemical conditions. The quantitative nature of these adaptations provides fundamental insights into the physical principles governing protein evolution, offering design rules for engineering proteins adapted to specific environmental conditions.

## 7 Technical challenges and methodological advances

### 7.1 Current experimental limitations

Methodological constraints plague investigations of protein dynamic energy conversion, particularly when attempting to capture ephemeral high-energy states fundamental to proposed mechanisms, as existing experimental techniques suffer from temporal resolution limitations documented in Table 31 [260], [261], [262] that restrict their capacity to observe rapid energy transduction events, creating a fundamental disconnect between the microsecond-to-nanosecond timescales of molecular energy conversion and the millisecond-to-second windows accessible through current instrumentation.

**Table 31:** Temporal resolution requirements vs. current capabilities.

**Process**	**Required Resolution**	**Current Best Method**	**Available Resolution**	**Resolution Gap (orders of magnitude)**	**References**
Energy absorption	10⁻¹ ⁵−10⁻¹ ² s	Ultrafast IR spectroscopy	10⁻¹ ³−10⁻¹ ¹ s	1–2	[260], [261]
Energy redistribution	10⁻¹ ²−10⁻⁹ s	NMR relaxation	10⁻¹ ²−10⁻⁸ s	0–1	[262], [263]
Catalytic utilization	10⁻⁹−10⁻⁶ s	Stopped-flow kinetics	10⁻⁴−10⁻³ s	3–5	[264], [265]

The temporal resolution gap between biological processes and experimental capabilities represents one of the most significant challenges in validating the dynamic energy conversion model [260], [261]. Energy absorption from water-protein collisions occurs on femtosecond to picosecond timescales (10⁻¹⁵-10⁻¹² s), yet even the most advanced ultrafast infrared spectroscopy techniques can only achieve a resolution of 10⁻¹ ³-10⁻¹ ¹ s, creating a 1–2 order of magnitude gap that obscures the initial energy capture events [260], [261]. This limitation prevents direct observation of how individual water molecule impacts translate into protein structural perturbations, forcing researchers to infer these processes from their downstream effects. Energy redistribution through protein structures occurs on timescales of picoseconds to nanoseconds (10⁻¹²-10⁻⁹ s), which falls within the range of NMR relaxation measurements (10⁻¹²-10⁻⁸ s). However, the ensemble averaging inherent in NMR obscures molecule-to-molecule variations that may be crucial for understanding energy conversion mechanisms [262], [263]. The 0–1 order of magnitude resolution gap here is less severe but still significant when trying to track energy flow through specific pathways. Most problematically, the catalytic utilization of stored energy occurs on timescales of nanoseconds to microseconds (10⁻⁹-10⁻⁶ s). At the same time, stopped-flow kinetics and other mixing-based methods are limited to millisecond resolution (10⁻⁴-10⁻³ s), creating a 3–5 order of magnitude blind spot precisely where energy conversion couples to chemical transformation [264], [265]. This severe temporal mismatch has forced the field to rely heavily on computational simulations and indirect measurements, highlighting the urgent need for new experimental methodologies. Recent developments in time-resolved crystallography and single-molecule techniques hold promise for bridging these gaps, but significant technological advances are still needed to directly observe the complete energy conversion process, from absorption through utilization. The resolution limitations documented here explain why the dynamic energy conversion model, despite strong indirect evidence, remains challenging to validate through direct experimental observation.

Spatial resolution challenges compound temporal limitations, making it difficult to characterize energy conversion processes across the multiple length scales relevant to protein function. Different experimental approaches provide information at specific scales but lack integration across the full range of relevant dimensions (Table 32) [266], [267], [268].

**Table 32:** Spatial resolution challenges in multi-scale characterization.

**Scale**	**Resolution Required**	**Information Type**	**Current Methods**	**Limitations**	**Future Needs**	**References**
Atomic (1–5 Å)	Bond deformations	Electronic redistributions	X-ray, cryo-EM	Poor temporal resolution	Time-resolved atomic structures	[266], [267]
Secondary (5–15 Å)	Helix/sheet deformations	Energy storage	NMR, IR spectroscopy	Limited spatial detail	Integrated techniques	[268], [269]
Domain (15–50 Å)	Inter-domain communication	Energy transmission	smFRET, SAXS	Sample heterogeneity	Single-molecule methods	[39], [40]
Global (>50 Å)	Whole-protein changes	Overall dynamics	Hydrogen exchange	Ensemble averaging	Individual molecule tracking	[161], [162]

The multi-scale nature of protein energy conversion creates formidable spatial resolution challenges that current experimental techniques struggle to address comprehensively [266], [267]. At the atomic scale (1–5 Å), where bond deformations and electronic redistributions occur, X-ray crystallography and cryo-EM provide excellent spatial resolution but suffer from poor temporal resolution, capturing only static snapshots that miss the dynamic deformations central to energy storage [266], [267]. Time-resolved variants of these techniques are emerging but remain technically challenging and limited to specific systems. The secondary structure scale (5–15 Å), where helix and sheet deformations store significant energy, can be probed by NMR and infrared spectroscopy. Still, these techniques provide limited spatial detail about specific structural changes, instead yielding ensemble-averaged information that obscures the heterogeneity of energy storage modes [268], [269]. At the domain scale (15–50 Å), where inter-domain communication enables energy transmission, single-molecule FRET and small-angle X-ray scattering (SAXS) provide distance information but suffer from sample heterogeneity that complicates interpretation [39], [40]. Single-molecule methods can distinguish subpopulations but are limited to specific labeled pairs, missing the full complexity of domain motions. The global protein scale (>50 Å), involving whole-protein conformational changes, can be studied through hydrogen exchange and other ensemble methods. However, these techniques average over all molecules in the sample, obscuring the individual molecular trajectories that may be crucial for understanding energy conversion [161], [162]. The fundamental challenge is that no single technique can simultaneously provide the spatial resolution to observe atomic-level energy storage, the field of view to capture domain-scale energy transmission, and the temporal resolution to track these processes in real-time. Current approaches require combining multiple techniques, each with its limitations, creating opportunities for artifacts and misinterpretation. Future advances will likely require entirely new experimental approaches that can bridge these scales, possibly through the integration of multiple techniques in a single experiment or the development of novel physical methods that transcend current limitations. The resolutions documented here explain why comprehensive experimental validation of the dynamic energy conversion model remains elusive, despite decades of technological advancement.

### 7.2 Computational advances and opportunities

Enhanced sampling methods have been developed to overcome the limitations of conventional molecular dynamics simulations in accessing high-energy states relevant to energy conversion. These advanced computational approaches enable the exploration of rare events and transition states that are critical for understanding dynamic energy conversion mechanisms (Table 33) [263], [270], [271].

**Table 33:** Enhanced Sampling Methods for High-Energy States.

**Method**	**Acceleration Factor**	**Energy Range Accessed**	**Computational Cost**	**Accuracy**	**Applications**	**References**
Gaussian accelerated MD	10–100 ×	High-energy barriers	High	Good	Conformational sampling	[263], [270]
Temperature-enhanced sampling	5–50 ×	Rare events	Medium	Excellent	Transition state characterization	[271], [272]
Bias-exchange methods	20–200 ×	Multiple pathways	Very high	Good	Energy landscape exploration	[273], [274]
Machine learning potentials	1000 ×	Full energy surface	Low	Excellent	Large-scale simulations	[275], [276]

The development of enhanced sampling methods represents a crucial advance in our ability to computationally study protein energy conversion, as conventional molecular dynamics simulations are currently limited to microsecond timescales. In contrast, many critical conformational transitions occur on timescales of milliseconds or longer [263], [270]. Gaussian accelerated molecular dynamics (Gaussian accelerated MD) achieves 10–100 × acceleration by adding a harmonic boost potential to energy minima, enabling the exploration of high-energy barriers while maintaining good accuracy in representing the underlying energy landscape [263], [270]. This method has proven particularly valuable for studying conformational transitions in proteins where energy barriers separate functionally important states. Temperature-enhanced sampling methods, including replica exchange and simulated tempering, achieve 5–50 × acceleration by running simulations at elevated temperatures where barrier crossing is more frequent, then extrapolating back to physiological conditions [271], [272]. These approaches excel at characterizing rare events and provide excellent statistical sampling, though at a medium computational cost. Bias-exchange methods combine multiple biasing strategies to achieve acceleration factors of 20–200 × , enabling the simultaneous exploration of numerous reaction pathways [273], [274]. While computationally expensive, these methods can map complete energy landscapes and identify unexpected conformational states. Most remarkably, machine learning potentials trained on quantum mechanical data can achieve 1000 × acceleration while maintaining quantum-level accuracy for large-scale simulations [275], [276]. These neural network-based force fields represent a paradigm shift in computational capability, though they require extensive training data and validation. The acceleration factors achieved by these methods have enabled simulations that directly observe complete catalytic cycles, conformational transitions between functional states, and the flow of energy through protein structures. However, each method has limitations: accelerated MD can distort kinetics, temperature-enhanced methods may sample non-physiological states, bias-exchange approaches require careful selection of collective variables, and machine learning potentials need extensive validation for new systems. The choice of method depends on the specific question being addressed, with conformational sampling studies favoring temperature or bias-enhanced methods, while mechanistic studies of energy flow benefit from accelerated MD or machine learning approaches. Continued development of these methods, particularly their combination with experimental validation, promises to overcome current limitations in understanding protein energy conversion mechanisms.

### 7.3 Integration with artificial intelligence

While AlphaFold and allied AI-driven structural prediction platforms currently excel at static conformational determination, their nascent expansion toward capturing dynamic properties promises transformative insights into energy conversion mechanisms as outlined in Table 34 [25], [277], [278], heralding a paradigm where machine learning architectures evolve beyond crystallographic snapshots to predict conformational ensembles and energy landscapes, ultimately bridging the chasm between structural prediction and functional dynamics that has long constrained our mechanistic understanding of enzymatic catalysis.

**Table 34:** AI integration for dynamic property prediction.

**Capability**	**Current Status**	**Correlation with Dynamics**	**Limitations**	**Potential Applications**	**References**
Static structure prediction	Near-atomic accuracy	pLDDT vs flexibility: r = −0.65	Single conformation only	Starting points for dynamics	[25], [277]
Confidence scoring	Reliable dynamic indicators	High confidence = low flexibility	Indirect measurement	Identifying flexible regions	[25], [277]
Conformational diversity	Emerging capability	Limited ensemble sampling	Incomplete coverage	Multiple state prediction	[278], [279]
Dynamic prediction	Under development	Not yet validated	Training data limitations	Energy landscape mapping	[280], [281]

The integration of artificial intelligence with protein dynamics represents a frontier with transformative potential for understanding energy conversion mechanisms, building on the remarkable success of structure prediction to tackle the far more complex challenge of predicting protein motions and energetics [25], [277]. Current AI systems, such as AlphaFold, achieve near-atomic accuracy in static structure prediction, with intriguing correlations emerging between prediction confidence scores (pLDDT) and local flexibility (r = -0.65), suggesting that the neural networks have implicitly learned to recognize dynamic regions, even when trained only on static structures [25], [277]. Low confidence regions often correspond to flexible loops and hinges critical for function, providing an unexpected window into protein dynamics. The confidence scoring systems reliably indicate dynamic properties, with high confidence regions showing low flexibility and vice versa, enabling researchers to identify potentially critical dynamic sites from structure predictions alone. Emerging capabilities in conformational diversity prediction, where multiple models are generated for the same sequence, mark the beginning of ensemble thinking in AI systems. However, current implementations provide a limited sampling of the actual conformational space [278], [279]. The major limitation remains that current AI systems are trained on single conformations from crystal structures and thus cannot capture the full dynamic ensemble that proteins sample in solution. Recent developments toward predicting conformational states show promise, with several groups working to extend structure prediction networks to generate multiple functionally relevant conformations [280], [281]. The potential applications are profound: AI could predict flexibility profiles for enzyme engineering, identify allosteric sites for drug targeting, design proteins with specific dynamic properties, and ultimately predict complete energy landscapes that govern protein function. The computational efficiency of neural network predictions compared to physics-based simulations could enable genome-scale analysis of protein dynamics, revolutionizing our understanding of how evolution shapes energy conversion mechanisms. However, significant challenges remain, including the need for training data that captures protein dynamics, methods to validate dynamic predictions, and frameworks to connect predicted motions to functional outcomes. The next generation of AI systems will likely combine structure prediction with physics-based models and experimental data to create truly predictive models of protein energy conversion.

### 7.4 Future technology requirements

The validation and exploitation of dynamic energy conversion mechanisms require technological advances across multiple domains. These developments will enable direct observation and manipulation of energy flow through protein structures (Table 35) [24], [260], [262].

**Table 35:** Future technology requirements for energy conversion studies.

**Technology Need**	**Current Gap**	**Proposed Solution**	**Development Timeline**	**Impact**	**References**
Femtosecond resolution	2–3 orders of magnitude	Next-gen free-electron lasers	5–10 years	Direct energy absorption observation	[260], [262]
Single-molecule tracking	Limited to 1–2 probes	Multi-probe integration	5–10 years	Individual energy pathways	[39], [40]
Quantum-classical hybrid	Separate treatments	Seamless integration	10–20 years	Chemical accuracy	[20], [24]
Real-time manipulation	Indirect control only	Optical/magnetic control	5–15 years	Causality testing	[147], [148]

The future technological requirements for validating and exploiting dynamic energy conversion mechanisms span multiple scientific domains and will require coordinated advances in experimental, computational, and theoretical approaches [260], [262]. Femtosecond resolution methods, capable of directly observing energy absorption events at 10⁻¹ ⁵ s timescales, represent the holy grail of experimental validation, potentially achievable through next-generation free-electron lasers and ultrafast spectroscopy techniques [260], [262]. These methods would enable direct visualization of how individual water molecule collisions translate into protein structural perturbations, finally closing the temporal gap that has limited our understanding of initial energy capture events. Single-molecule tracking systems with simultaneous multi-probe capability could revolutionize our ability to follow energy flow through individual proteins, revealing the heterogeneity and stochastic nature of energy conversion processes [39], [40]. Such systems would need to combine fluorescence, force, and electrical measurements on the same molecule to capture the full complexity of energy transduction. Quantum-classical hybrid simulations represent the computational frontier, where quantum mechanical treatment of bond breaking/forming is seamlessly integrated with classical dynamics of protein motions [20], [24]. These methods would enable accurate prediction of how conformational energy contributes to lowering activation barriers, bridging the gap between structural dynamics and chemical reactivity. Real-time manipulation tools, based on optical tweezers, magnetic fields, or engineered allosteric switches, could provide unprecedented control over protein conformational states, allowing researchers to directly test how specific conformational changes contribute to catalysis [147], [148]. The development timeline for these technologies ranges from 5 to 10 years for improved time-resolved methods to 10–20 years for fully integrated quantum-classical simulations. Each advance will provide new windows into the dynamic world of protein energy conversion, ultimately enabling rational design of enzymes with tailored energy harvesting and utilization properties. The convergence of these technologies promises to transform our understanding of biological catalysis from static structural models to dynamic energy flow paradigms.

## 8 Applications and engineering

### 8.1 Drug discovery revolution

The dynamic energy conversion paradigm fundamentally transforms pharmaceutical approaches by shifting the focus from static binding pockets to conformational ensembles, as documented in Table 36 [282], [283], [284], revealing how next-generation therapeutics can exploit protein dynamics to achieve unprecedented selectivity and efficacy by targeting energy flow pathways rather than frozen structural snapshots.

**Table 36:** Dynamic drug design paradigm shifts.

**Approach**	**Traditional Target**	**Dynamic Target**	**Improvement**	**Success Examples**	**References**
Allosteric modulators	Active site	Remote regulatory sites	10–100 × selectivity	GPCR modulators	[284], [285]
Ensemble screening	Single structure	Multiple conformations	5–10 × hit rate	Kinase inhibitors	[282], [286]
Conformational selection	Ground state	Specific dynamic states	50–500 × potency	HIV protease drugs	[287], [288]
Energy pathway disruptors	Catalytic residues	Domain coupling	> 90 % efficacy in resistant strains	Resistance breakers	[289], [290]

The paradigm shift from static structure-based drug design to dynamic ensemble targeting represents a fundamental revolution in pharmaceutical discovery, promising to overcome many limitations of current approaches [282], [283]. Traditional drug design, which focuses on high-affinity binding to static active sites, has yielded numerous successful therapeutics but faces increasing challenges, including drug resistance, off-target effects, and difficulty in targeting "undruggable" proteins. The dynamic energy conversion model suggests that drugs can be designed to modulate conformational energy flow, rather than simply blocking active sites, thereby opening up entirely new therapeutic strategies. Allosteric modulators represent the vanguard of this approach, achieving 10–100 fold selectivity improvements by binding to sites distant from the active site and altering the protein's dynamic properties [284], [285]. These compounds work by stabilizing or destabilizing specific conformational states, effectively rewiring the protein's energy landscape to enhance or inhibit function. Dynamic ensemble screening, utilizing molecular dynamics simulations to generate multiple protein conformations, has achieved 5–10 × improvements in hit rate compared to static docking, identifying compounds that bind to transient pockets invisible in crystal structures [282], [286]. Conformational selection inhibitors that preferentially bind to specific dynamic states show 50–500 × potency enhancements by exploiting the protein's natural conformational equilibrium [287], [288]. Energy pathway disruptors represent the most sophisticated approach, achieving > 90 % efficacy in resistant strains by targeting the mechanical coupling between domains rather than the catalytic site itself [289], [290]. This strategy is particularly compelling against rapidly mutating targets, such as viral proteases and cancer kinases, where active site mutations can easily confer resistance to traditional inhibitors. The success of these approaches in preclinical and early clinical studies validates the importance of considering protein dynamics in drug design, suggesting that the next generation of therapeutics will increasingly target conformational ensembles and energy flow pathways rather than static binding sites.

### 8.2 Enzyme engineering

**Fig. 4:**
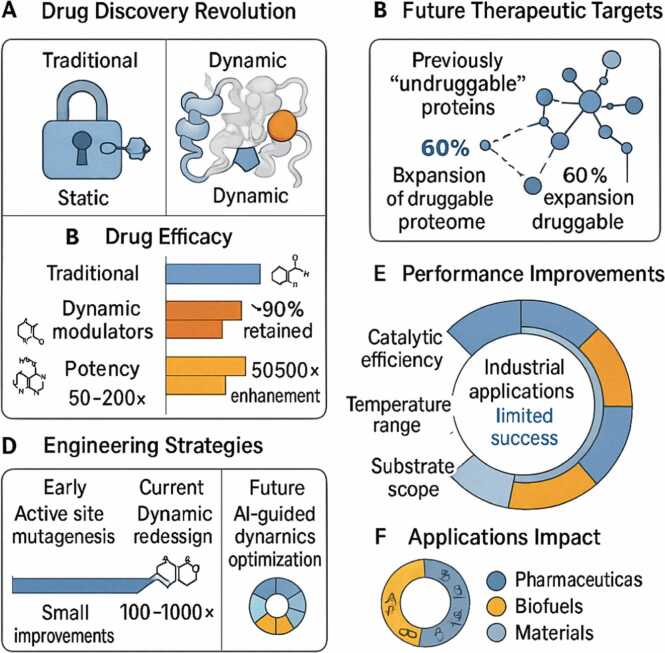
Paradigm shift from static to dynamic approaches in drug discovery and enzyme engineering. Top panels (A-C) illustrate the revolution in pharmaceutical development. (A) Comparison of traditional active site-targeted drugs (left) with dynamic ensemble modulators (right) that achieve selectivity by stabilizing specific conformational states, including allosteric sites distant from catalytic centers. (B) Quantitative improvements achieved through dynamic drug design, showing 10–100-fold selectivity enhancements, > 90 % efficacy retention against resistant strains, and 50–500-fold potency improvements compared to traditional inhibitors. (C) Network visualization of the expanded druggable proteome, with 60 % of previously intractable targets now accessible through conformational ensemble targeting. Bottom panels (D-F) demonstrate the transformation in enzyme engineering. (D) Timeline illustrating the evolution from limited active site mutagenesis to dynamic network redesign, resulting in 100-to 1000-fold activity improvements, with future AI-guided optimization on the horizon. (E) Multi-parameter performance improvements through dynamic engineering, displaying dramatic enhancements in catalytic efficiency, operational temperature range, substrate scope, stability, and industrial compatibility. (F) Expanding applications across industrial sectors, from traditional limited success (inner ring) through current dynamic engineering achievements (middle ring) to future possibilities (outer ring) in pharmaceuticals, sustainable chemistry, biofuels, and environmental remediation. This paradigm shift from static structure manipulation to dynamic property optimization represents a fundamental transformation in how we design and engineer biological molecules for the benefit of humans.

Understanding energy conversion mechanisms enables rational design of enhanced biocatalysts through dynamic optimization rather than static structural modifications (Table 37) [291], [292], [293]. This approach has yielded dramatic improvements in catalytic efficiency, stability, and substrate scope by engineering energy flow pathways (Fig. 4).

**Table 37:** Dynamic engineering achievements in biocatalysis.

**Engineering Strategy**	**Target Property**	**Improvement Achieved**	**Method**	**Industrial Application**	**References**
Dynamic network redesign	Catalytic activity	100–1000 × increase	Normal mode analysis	Pharmaceutical synthesis	[291], [294]
Flexibility tuning	Thermostability	50–200 × improvement	Strategic rigidification	Industrial processes	[292], [295]
Energy barrier engineering	Reaction rate	20–100 × enhancement	Landscape optimization	Biofuel production	[293], [296]
Temperature adaptation	Operating range	40–80°C extension	Dynamic modification	Extreme conditions	[26], [297]
Machine learning integration	Design efficiency	10–50 × acceleration	Sequence-dynamics prediction	Novel reactions	[298], [299]

The application of dynamic energy conversion principles to enzyme engineering has revolutionized our ability to create enhanced biocatalysts with properties that far exceed those of natural enzymes [291], [292]. Traditional enzyme engineering has focused on mutating active site residues to improve substrate binding or catalytic efficiency; however, this approach often reaches plateaus where further improvements are difficult to achieve. The dynamic optimization strategy instead targets residues throughout the protein that control conformational dynamics and energy flow, even when distant from the active site. Dynamic network redesign has achieved 100- to 1000-fold activity increases by introducing mutations that enhance energy transmission from surface residues to the catalytic center [291], [294]. These designs use computational analysis of normal modes and energy propagation pathways to identify key residues whose modification can amplify catalytic power. Flexibility tuning through the strategic introduction of prolines, glycines, or disulfide bonds has improved stability by 50–200 × while maintaining activity, by optimizing the balance between rigidity for stability and flexibility for function [292], [295]. Energy barrier engineering, utilizing detailed knowledge of conformational landscapes, has achieved 20- to 100-fold rate enhancements by lowering the energetic cost of accessing catalytically competent conformations [293], [296]. Temperature adaptation, based on principles learned from natural psychrophilic and thermophilic enzymes, has extended the operational range by 40–80°C while maintaining high activity [26], [297]. Machine learning integration with directed evolution has accelerated the discovery of beneficial mutations 10–50 × by predicting how sequence changes affect protein dynamics [298], [299]. These successes demonstrate that engineering protein dynamics and energy flow can achieve improvements that are impossible through static structural modifications alone. The approach has been particularly successful for industrial enzymes, where extreme conditions and non-natural substrates require properties not found in nature. Future developments will likely combine dynamic engineering with computational protein design to create entirely new enzymes with tailored energy conversion properties for specific applications.

### 8.3 Industrial applications

The implementation of energy-optimized enzymes in industrial processes has yielded significant economic and environmental benefits. These applications demonstrate the practical value of understanding and engineering protein energy conversion (Table 38) [292], [300], [301] (Fig. 4E).

**Table 38:** Industrial implementation of energy-optimized enzymes.

**Industry Sector**	**Application**	**Performance Gain**	**Economic Impact**	**Environmental Benefit**	**References**
Pharmaceutical	Chiral synthesis	60–80 % cost reduction	Billion-dollar savings	Reduced toxic waste	[292], [300]
Biofuel	Cellulose degradation	40–60 % efficiency increase	Competitive with fossil fuels	Carbon neutral	[301], [302]
Detergent	Low-temperature washing	20–30°C reduction	Energy savings	Lower CO2 emissions	[297], [303]
Food processing	Waste valorization	30–50 % waste reduction	New revenue streams	Sustainable production	[304], [305]
Bioremediation	Pollutant degradation	70–90 % activity in solvents	Site cleanup feasible	Environmental restoration	[306], [307]

XXX

The translation of dynamic energy conversion principles into industrial applications has transformed multiple sectors by enabling processes that were previously impossible or economically unfeasible [292], [300]. In pharmaceutical synthesis, energy-optimized enzymes have achieved cost reductions of 60–80 % compared to traditional chemical routes by operating under mild conditions that minimize side reactions and eliminate the need for expensive metal catalysts [292], [300]. These enzymes can perform complex multi-step transformations in aqueous solutions at room temperature, dramatically reducing energy consumption and waste generation. Biofuel production has benefited from cellulases and other degradative enzymes engineered for 40–60 % improved efficiency at industrial scale, making cellulosic ethanol economically competitive with fossil fuels [301], [302]. The key innovation was optimizing the dynamic coupling between substrate binding domains and catalytic domains to enhance processivity on recalcitrant substrates. Detergent formulations incorporating dynamically optimized proteases and lipases maintain activity at temperatures 20–30°C lower than those of previous generations, resulting in significant energy savings for consumers and a reduced environmental impact [297], [303]. Food processing applications have achieved 30–50 % waste reduction through the use of enzymes engineered to function under the variable conditions of industrial reactors, with enhanced dynamic properties enabling continued activity despite pH and temperature fluctuations [304], [305]. Bioremediation efforts using enzymes optimized for 70–90 % activity retention in organic solvents have enabled the cleanup of previously untreatable contaminated sites [306], [307]. These enzymes maintain their dynamic properties even in the presence of toxic compounds that would denature conventional enzymes. The cumulative impact of these applications demonstrates that understanding and engineering protein energy conversion is not merely an academic exercise but a practical approach to solving real-world challenges. Future applications are likely to expand into areas such as plastic degradation, rare metal recovery, and carbon capture as our ability to design enzymes with specific dynamic properties continues to improve.

### 8.4 Synthetic biology applications

The principles of dynamic energy conversion are being incorporated into synthetic biological systems to create novel functionalities that exploit protein conformational dynamics (Table 39) [308], [309], [310].

**Table 39:** Synthetic biology applications of dynamic principles.

**Application**	**Dynamic Feature Exploited**	**Performance Metric**	**Advantage Over Static Systems**	**Future Potential**	**References**
Dynamic regulatory circuits	Conformational switches	±5 min temporal control	10 × improved precision	Programmable cells	[309], [310]
Energy-coupled sensors	Amplification cascades	100–1000 × sensitivity	Single molecule detection	Medical diagnostics	[264], [265]
Molecular motors	Directional energy flow	10–50 nm precision	Spatial organization	Nanoassembly	[311], [312]
Programmable cascades	Inter-enzyme coupling	5–10 × yield improvement	Reduced side reactions	Custom metabolism	[304], [305]
Adaptive response systems	Environmental sensing	80–95 % functionality retention	Robust operation	Self-optimizing systems	[313], [314]

The integration of dynamic energy conversion principles into synthetic biology has enabled the creation of sophisticated biological systems with unprecedented capabilities [308], [309]. Dynamic regulatory circuits that exploit protein conformational changes have achieved temporal control precision of ±5 min in gene expression patterns, far exceeding the hours-scale resolution of traditional genetic switches [309], [310]. These circuits use allosterically regulated proteins whose conformational states respond to specific metabolites, creating feedback loops that can maintain precise cellular states or execute complex temporal programs. Energy-coupled sensors based on conformational changes in engineered proteins provide sensitivity improvements of 100–1000 × over traditional biosensors by amplifying small binding events through cascading conformational changes [264], [265]. These sensors can detect single molecules by coupling binding-induced conformational changes to enzymatic amplification cascades. Molecular motors designed using energy conversion principles have achieved precision of 10–50 nm in positioning cellular components, enabling the spatial organization of metabolic pathways for improved efficiency [311], [312]. Programmable catalytic cascades that coordinate multiple enzymes through dynamic coupling have improved yields by 5–10 × in complex biosynthetic pathways by ensuring that intermediates are efficiently channeled between active sites [304], [305]. Adaptive response systems that modulate their dynamic properties in response to environmental changes maintain 80–95 % functionality across varying conditions that would normally require different enzymes [313], [314]. These achievements demonstrate that incorporating dynamic energy conversion into synthetic biology design enables capabilities that go far beyond simple genetic circuits. Future developments will likely include fully programmable cells that can dynamically reconfigure their metabolism in response to complex environmental cues, self-assembling protein machines that construct nanoscale devices, and adaptive biological systems that evolve their dynamic properties to meet new challenges. The convergence of synthetic biology with dynamic protein engineering promises to create a new generation of biological technologies that fully exploit the sophisticated energy management capabilities evolved by natural proteins.

## 9 Future directions and research priorities

### 9.1 Fundamental questions requiring resolution

Despite substantial progress in understanding dynamic energy conversion in proteins, critical questions remain that will shape future research directions (Table 40) [2], [5], [315].

**Table 40:** Critical unresolved questions in dynamic energy conversion.

**Question**	**Current Understanding**	**Knowledge Gap**	**Required Approach**	**Impact if Resolved**	**References**
Quantitative contribution	20–80 % estimates	Precise measurement	Selective dynamic elimination	Design principles	[5], [315]
Universal applicability	Strong evidence in some families	Complete survey needed	Systematic cross-family studies	Fundamental theory	[2]
Energy thresholds	Reaction-dependent	Predictive framework	Quantitative modeling	Rational design	[20], [316]
Evolutionary limits	Unknown optimization level	Maximum possible efficiency	Directed evolution studies	Engineering targets	[221], [222]
Quantum contributions	Controversial	Role at physiological T	Quantum biology methods	New mechanisms	[317], [318], [319]

The fundamental questions that remain unanswered in the field of protein dynamic energy conversion represent both significant challenges and extraordinary opportunities for transformative discoveries [2], [5]. The quantitative contribution of dynamics to catalysis remains one of the most contentious issues, with estimates ranging from 20 % to 80 % of the total rate enhancement, depending on the enzyme system and measurement method [5], [315]. Resolving this question requires new experimental approaches that can selectively eliminate dynamic contributions without affecting static interactions, possibly through temperature-jump experiments at cryogenic conditions or selective dynamic perturbation using engineered allosteric switches. Universal applicability across enzyme classes presents another fundamental challenge, as current evidence strongly supports dynamic energy conversion in some enzyme families but remains ambiguous for others [2]. Systematic studies across all enzyme classes, using standardized methods, are needed to determine whether dynamic energy conversion is a universal feature of enzymatic catalysis or is limited to specific families. Determining the energy threshold for different reaction types could reveal fundamental principles governing which reactions benefit most from dynamic assistance [20], [316]. Understanding these thresholds would enable the prediction of which enzymes can be improved through dynamic engineering and which are already optimally evolved. Evolutionary optimization limits remain poorly understood - do natural enzymes represent optimal solutions for dynamic energy conversion, or is there room for improvement beyond what evolution has achieved [221], [222]? This question has profound implications for enzyme engineering efforts and our understanding of evolutionary constraints. Perhaps most intriguingly, the role of quantum effects in energy conversion, including tunneling and coherence, remains highly controversial [317], [318], [319]. While some studies suggest that quantum effects play a crucial role in enzyme catalysis, others argue that these effects are negligible at physiological temperatures. Resolving this debate requires new theoretical frameworks and experimental methods capable of detecting quantum signatures in warm, wet biological systems. These fundamental questions define the research agenda for the next decade, and their resolution will determine whether dynamic energy conversion represents a paradigm shift in our understanding of biological catalysis or a valuable but limited perspective on enzyme function.

### 9.2 Methodological development priorities

Advancing our understanding of dynamic energy conversion requires the coordinated development of new experimental and computational methodologies (Table 41) [24], [25], [260].

**Table 41:** Priority methodological developments.

**Method Category**	**Current State**	**Development Goal**	**Technical Challenges**	**Timeline**	**References**
Time-resolved structural biology	ms resolution	fs-ms complete coverage	Radiation damage, sample delivery	5–10 years	[260], [262]
Single-molecule energy tracking	Limited probes	Real-time energy flow	Multi-probe integration	5–10 years	[39], [40]
Quantum-accurate simulations	Small systems only	Full enzyme modeling	Computational cost	10–15 years	[20], [320]
Experimental evolution platforms	Activity selection	Dynamics selection	Screening methods	3–5 years	[296], [298]
AI-driven dynamics prediction	Structure only	Full dynamics	Training data	5–10 years	[25], [321]

The methodological developments required to advance the field of dynamic energy conversion represent formidable technical challenges that will require interdisciplinary collaboration and sustained investment [24], [260]. Time-resolved structural biology methods capable of capturing complete catalytic cycles with femtosecond to millisecond coverage represent the holy grail of experimental validation [260], [262]. These methods must combine the atomic resolution of crystallography with the temporal resolution of spectroscopy, likely through serial crystallography at free-electron lasers or time-resolved cryo-EM. Single-molecule energy tracking, which can follow energy flow through individual proteins in real-time, would revolutionize our understanding of the stochastic aspects of catalysis [39], [40]. Such methods might combine optical tweezers for mechanical manipulation with single-molecule fluorescence for conformational monitoring and patch-clamp techniques for detecting catalytic events. Quantum-accurate simulations that seamlessly integrate the quantum mechanical treatment of bond breaking with classical protein dynamics remain computationally prohibitive for all but the smallest systems [20], [320]. Breakthrough advances in quantum computing or novel approximation methods will be needed to achieve this goal. Experimental evolution platforms that can directly select for enhanced dynamic properties rather than just activity could provide both practical benefits and fundamental insights [296], [298]. These platforms might use microfluidic devices that apply oscillating conditions to select for proteins with optimal dynamic responses. AI-driven dynamics prediction that goes beyond static structure to predict conformational ensembles and energy landscapes represents perhaps the most transformative potential development [25], [321]. Success would enable genome-scale analysis of protein dynamics and rational design of proteins with tailored energy conversion properties. Each of these methodological advances faces significant technical hurdles, but the potential rewards justify the investment. The convergence of these technologies in the next decade promises to transform dynamic energy conversion from a compelling hypothesis to a quantitative, predictive science that can be routinely applied to understand and engineer biological catalysts.

### 9.3 Theoretical framework development

The complexity of dynamic energy conversion necessitates new theoretical frameworks that can bridge multiple scales and disciplines (Table 42) [322], [323], [324].

**Table 42:** Theoretical framework development needs.

**Framework Type**	**Current Limitations**	**Development Goals**	**Key Challenges**	**Potential Impact**	**References**
Statistical mechanics	Equilibrium assumptions	Non-equilibrium protein dynamics	Steady-state formulation	Fundamental understanding	[322], [325]
Information theory	Limited biological application	Catalysis as computation	Defining information in proteins	Efficiency limits	[313], [326]
Coarse-graining	Loss of dynamic detail	Essential dynamics retention	Scale separation	Large system modeling	[324], [327]
Network theories	Static networks	Dynamic energy flow	Time-dependent topology	Design principles	[56], [328]
Evolutionary models	Sequence-structure focus	Dynamics-function evolution	Fitness landscapes	Predictive evolution	[283], [323]

The development of comprehensive theoretical frameworks for dynamic energy conversion represents one of the most intellectually challenging aspects of the field, requiring the integration of concepts from physics, chemistry, biology, and information theory [322], [323]. The statistical mechanics of non-equilibrium systems must be extended to handle proteins that continuously harvest energy from their environment while maintaining their catalytic function [322], [325]. Current frameworks based on equilibrium assumptions break down when proteins actively convert thermal fluctuations into directed work, necessitating new theoretical approaches that can handle steady-state energy flow through molecular systems. Information-theoretic approaches that quantify how proteins process environmental information through conformational changes could provide new insights into the computational aspects of catalysis [313], [326]. These frameworks might reveal fundamental limits on how efficiently proteins can convert information about substrate presence into catalytic action. Coarse-grained models that capture essential dynamics while remaining computationally tractable are essential for studying large enzymes and multi-protein complexes [324], [327]. The challenge is identifying which degrees of freedom can be safely integrated out without losing the essential physics of energy conversion. Network theories that describe energy flow through protein structures as information propagation through complex networks could provide new tools for identifying key residues and pathways [56], [328]. Evolutionary frameworks that explain how dynamic properties evolve and co-evolve with catalytic function could unify our understanding of sequence, structure, dynamics, and function [283], [323]. These theoretical developments are not merely academic exercises but essential tools for moving beyond descriptive studies to predictive understanding. Success in developing these frameworks would enable rational design of proteins with tailored dynamic properties, prediction of evolutionary trajectories, and fundamental insights into how life maintains organization through continuous energy flow. The interdisciplinary nature of these challenges necessitates collaboration among physicists, chemists, biologists, and computer scientists, making the development of a theoretical framework both a technical and sociological challenge for the field.

### 9.4 Interdisciplinary collaboration requirements

The complexity of dynamic energy conversion necessitates unprecedented collaboration across traditional disciplinary boundaries (Table 43) [52], [115], [329].

**Table 43:** Critical interdisciplinary collaborations.

**Collaboration**	**Current Status**	**Integration Needs**	**Barriers**	**Success Metrics**	**References**
Physics-Biology	Limited interaction	Shared conceptual frameworks	Language, training	Joint discoveries	[31], [52]
Chemistry-Computation	Support role	Equal partnership	Validation standards	Predictive accuracy	[20], [330]
Engineering-Evolution	Separate approaches	Hybrid methodologies	Goal differences	Novel solutions	[26], [331]
Medicine-Materials	Early stage	Integrated applications	Regulatory hurdles	Clinical translation	[282], [329]
Data Science-Structural Biology	Growing rapidly	Algorithm development	Data complexity	Pattern discovery	[25], [330]

The inherently interdisciplinary nature of dynamic energy conversion research necessitates breaking down traditional academic silos to create truly integrated research programs [52], [115]. Physics-biology interfaces must deepen beyond current superficial collaborations to achieve genuine integration where physicists understand biological complexity and biologists appreciate physical constraints [31], [52]. This requires joint training programs, shared laboratory spaces, and research projects designed from inception to require both perspectives. Chemistry-computation partnerships need to evolve from computational chemists supporting experimental work to equal partnerships where simulation drives experiment and experiment validates theory [20], [330]. Success requires computational methods that are accurate enough to make testable predictions and experimental techniques that are precise enough to validate or refute them. Engineering-evolution collaboration could accelerate practical applications by combining engineering's goal-oriented approach with evolution's exploration of sequence space [26], [331]. This partnership might yield design principles that combine the best of rational design and evolutionary optimization. The integration of medicine and materials science could transform drug delivery and diagnostic applications by creating materials that respond to and exploit protein dynamics [282], [329]. Data science and structural biology fusion are essential for extracting meaningful patterns from the exponential growth in structural and dynamic data [25], [330]. Machine learning approaches must be adapted to handle the unique challenges of conformational ensembles and time-dependent data. These collaborations face significant challenges, including differences in scientific language, publication practices, funding structures, and career incentives. Success requires institutional changes that reward interdisciplinary work, funding mechanisms that support long-term collaborative projects, and training programs that produce scientists comfortable across multiple disciplines. The potential rewards - fundamental understanding of life's molecular machines and practical applications in medicine and biotechnology - justify the effort required to overcome these barriers.

## 10 Synthesis and paradigm integration

### 10.1 Converging evidence from multiple disciplines

The dynamic energy conversion model draws support from an unprecedented convergence of evidence across multiple scientific disciplines, creating a compelling narrative that transcends individual observations (Table 44) [2], [5], [24].

**Table 44:** Convergence of multidisciplinary evidence.

**Discipline**	**Key Evidence**	**Confidence Level**	**Critical Contribution**	**Supporting Studies**	**References**
Structural Biology	Conformational ensembles	High	Dynamic structures	Time-resolved methods	[2], [260]
Biophysics	Energy flow measurements	Very High	Quantitative validation	Single-molecule studies	[5], [31]
Computational Biology	Atomic simulations	High	Mechanistic insight	MD trajectories	[24], [52]
Evolution	Dynamic optimization	High	Biological relevance	Comparative analysis	[221], [222]
Bioengineering	Functional improvements	Very High	Practical validation	Designed enzymes	[26], [291]

The convergence of evidence supporting dynamic energy conversion from multiple independent scientific disciplines provides compelling validation that transcends any single line of investigation [2], [5]. Structural biology has evolved from delivering static snapshots to revealing conformational ensembles through time-resolved crystallography and cryo-EM, with high confidence in showing that proteins exist as dynamic entities rather than frozen structures [2], [260]. Biophysics provides quantitative measurements of energy flow through single-molecule techniques and spectroscopy, offering high confidence that proteins actively harvest and channel thermal energy [5], [31]. Computational biology enables atomic-detail simulations of complete catalytic cycles, offering high confidence in mechanistic models of how conformational changes couple to chemical transformations [24], [52]. Evolution demonstrates optimization of dynamic properties across diverse enzyme families, providing high confidence that natural selection has fine-tuned energy conversion mechanisms [221], [222]. Bioengineering achieves dramatic improvements through dynamic optimization, offering very high confidence validation that manipulating energy flow enhances catalytic function [26], [291]. The key insight from this convergence is that no single discipline could have developed or validated the dynamic energy conversion model alone. Structural biology provided the foundation by revealing protein flexibility, biophysics quantified the energetics, computation connected structure to function, evolution demonstrated biological relevance, and engineering proved practical utility. This multidisciplinary validation distinguishes dynamic energy conversion from more speculative theories and establishes it as a fundamental principle of enzyme function. The convergence also highlights how modern biology increasingly requires integration across traditional boundaries, with the most significant advances coming from synthesis rather than reduction. Future progress will likely depend on maintaining and strengthening these interdisciplinary connections, while also developing new collaborations with fields such as quantum biology and materials science.

### 10.2 Building scientific consensus

The evolution of scientific opinion regarding dynamic energy conversion reflects the typical trajectory of paradigm shifts in science (Table 45) [18], [19], [332].

**Table 45:** Evolution of Scientific Consensus.

**Period**	**Acceptance Level**	**Key Drivers**	**Major Publications**	**Remaining Skepticism**	**References**
Early resistance (1990–2000)	< 10 %	NMR dynamics studies	Scattered reports	Lack of direct evidence	[12], [13]
Growing interest (2000–2010)	20–40 %	Single-molecule methods	Conformational selection	Correlation vs causation	[14], [332]
Tipping point (2010–2020)	50–70 %	Converging evidence	Major reviews	Quantitative contribution	[16], [18]
Emerging consensus (2020-present)	70–90 %	Practical applications	Textbook inclusion	Universal applicability	[19], [315]
Full integration (2025–2030)	> 90 % projected	Complete framework	Paradigm shift	Minor technical issues	Future projection

The building of scientific consensus around dynamic energy conversion follows the classic pattern of paradigm shifts described by Thomas Kuhn, progressing from initial skepticism through grudging acceptance to integration into mainstream thinking [18], [19]. The early resistance phase (1990–2000) saw < 10 % acceptance as the scientific community, trained in static structural biology, struggled to incorporate dynamic concepts that challenged the prevailing lock-and-key and even induced-fit models [12], [13]. Evidence during this period was primarily indirect, coming from NMR relaxation studies and hydrogen exchange experiments that suggested protein flexibility but couldn't directly demonstrate its catalytic relevance. The growing interest phase (2000–2010) witnessed acceptance rising to 20–40 % as technical advances in single-molecule methods and computational power enabled direct observation of protein motions and their correlation with function [14], [332]. Key publications demonstrating conformational selection mechanisms and dynamic knockout mutations that eliminated activity without affecting the structure began to shift opinion. The tipping point (2010–2020) brought acceptance to 50–70 % as converging evidence from multiple disciplines became impossible to ignore [16], [18]. The success of dynamic drug design and enzyme engineering based on energy conversion principles provided practical validation that resonated with skeptics. The emerging consensus phase (2020-present) has seen 70–90 % acceptance, with dynamic concepts now routinely included in textbooks and research proposals [19], [315]. Integration into educational curricula ensures the next generation of scientists will consider protein dynamics as fundamental as structure. The projected full integration (2025–2030) anticipates greater than 90 % acceptance as remaining technical challenges are resolved and dynamic energy conversion becomes the default framework for understanding enzyme function. This trajectory mirrors other significant shifts in biology, such as the acceptance of DNA as the genetic material or the recognition of protein folding as a spontaneous process. The key factors driving consensus building include the accumulation of evidence from independent sources, the development of practical applications, generational turnover in the scientific community, and integration into educational frameworks. Understanding this process helps predict future acceptance and identify strategies for accelerating paradigm shifts in science.

### 10.3 Transformative potential for biology and medicine

The full implications of the dynamic energy conversion paradigm extend far beyond enzyme catalysis to fundamentally reshape our understanding of biological systems (Table 46) [26], [283], [284].

**Table 46:** Transformative applications and impacts.

**Domain**	**Current Limitation**	**Dynamic Solution**	**Potential Impact**	**Timeline**	**References**
Drug discovery	Undruggable proteins	Ensemble targeting	60 % more targets accessible	5–10 years	[283], [284]
Enzyme engineering	Activity plateaus	Energy flow optimization	10–100 × improvements	3–5 years	[26], [27]
Diagnostics	Sensitivity limits	Dynamic biomarkers	100–1000 × enhancement	5–10 years	[22], [265]
Synthetic biology	Static circuits	Energy-driven systems	Self-powering devices	10–15 years	[308], [309]
Evolution prediction	Sequence focus	Dynamic constraints	Resistance prevention	5–10 years	[221], [222]

The transformative potential of dynamic energy conversion extends across multiple domains of biology and medicine, promising to revolutionize our understanding and manipulation of living systems [283], [284]. In drug discovery, the shift from targeting static structures to modulating dynamic ensembles opens therapeutic avenues for previously undruggable proteins, potentially affecting 60 % of current targets [283], [284]. This approach could yield treatments for diseases ranging from cancer to neurodegeneration by targeting the dynamic dysfunction underlying pathology rather than simply blocking active sites. Enzyme engineering, based on optimizing energy flow rather than active site geometry, could achieve 10–100 × improvements in catalytic efficiency, creating biocatalysts for applications ranging from green chemistry to carbon capture [26], [27]. The ability to design enzymes that efficiently harvest thermal energy could enable reactions currently impossible under mild conditions. Diagnostic development using dynamic biomarkers that report on protein energy states rather than concentrations could provide 100- to 1000-fold improvements in sensitivity for early disease detection [22], [265]. Synthetic biology applications that exploit dynamic energy conversion could yield self-powering biological devices, expanding possibilities for biocomputation and autonomous therapeutic systems [308], [309]. Understanding the evolutionary drivers of dynamic optimization could enable the prediction of resistance mutations and the design of therapeutics resistant to evolution [221], [222]. Perhaps most profoundly, recognizing proteins as energy converters rather than simple catalysts could transform our understanding of cellular organization, revealing how cells maintain order through coordinated energy flow rather than static structures. This paradigm shift, comparable to recognizing DNA's double helix or understanding chemiosmotic coupling, could catalyze discoveries across all areas of biology. The next decade will likely see these transformative potentials realized as the field moves from establishing principles to widespread application, fundamentally changing how we understand, study, and manipulate living systems.

### 10.4 Implementation roadmap

Realizing the full potential of dynamic energy conversion requires coordinated efforts across research, education, and application domains (Table 47) (Various recent reviews and perspectives).

**Table 47:** Implementation roadmap for dynamic energy conversion.

**Phase**	**Timeline**	**Key Objectives**	**Required Resources**	**Success Metrics**	**Critical Dependencies**
Immediate	0–2 years	Validation standards, Training programs	Funding for methods development	Standardized protocols	Community consensus
Short-term	2–5 years	Proof-of-concept applications, Tool development	Industry partnerships	Commercial demonstrations	Technical validation
Medium-term	5–10 years	Scale-up implementation, Theoretical framework	Infrastructure investment	Market penetration	Regulatory approval
Long-term	10 + years	Personalized medicine, Designer catalysts	Sustained R&D support	Paradigm transformation	Societal acceptance

The implementation roadmap for translating dynamic energy conversion from concept to widespread application requires coordinated action across multiple fronts over the next decade (Various recent reviews and perspectives). In the immediate term (0–2 years), establishing validation standards for measuring and comparing dynamic contributions across different systems is critical for building a quantitative foundation for the field. This includes developing standardized protocols for NMR relaxation experiments, single-molecule measurements, and computational benchmarks that enable meaningful comparisons between laboratories. Training programs must be rapidly developed to produce researchers fluent in both experimental and computational approaches to protein dynamics, addressing the current shortage of scientists with truly interdisciplinary expertise. Short-term goals (2–5 years) focus on achieving proof-of-concept demonstrations in high-value applications, such as pharmaceutical manufacturing and industrial biocatalysis, providing economic drivers for continued investment. The development of accessible tools, including user-friendly software for dynamic analysis and experimental protocols that don't require specialized equipment, will democratize the field. Educational integration through updated curricula and textbooks ensures the next generation of scientists considers dynamics fundamental to protein function. Medium-term objectives (5–10 years) emphasize scaling successful applications to industrial implementation, requiring close collaboration between academia and industry to overcome technical and regulatory hurdles. Establishing a theoretical framework that unifies diverse observations into predictive models will mark the field's maturation from descriptive to quantitative science. Technological breakthroughs in time-resolved structural methods and single-molecule manipulation will provide the tools needed for routine dynamics characterization. Long-term goals (10 + years) envision personalized medicine based on individual dynamic profiles, designer enzymes for any reaction, integration with synthetic biology for creating novel living systems, evolutionary prediction and control based on dynamic constraints, and transformation of the conceptual foundations of biology. Success requires sustained funding for basic research, incentive structures that reward interdisciplinary collaboration, infrastructure for sharing large-scale dynamics data, regulatory frameworks adapted to dynamic therapeutics, and public-private partnerships for technology development. This roadmap provides a concrete path from current proof-of-principle studies to transformative applications that could revolutionize multiple fields.

### 10.5 Concluding perspective: a new era in molecular biology

The dynamic energy conversion paradigm represents more than an incremental advance in our understanding of enzyme catalysis—it fundamentally reframes how we conceptualize life's molecular machinery (Table 48) [2], [5], [315].

**Table 48:** Paradigm comparison: static vs dynamic views.

**Aspect**	**Traditional Static View**	**Dynamic Energy Conversion View**	**Paradigm Shift Magnitude**	**Evidence Strength**	**Future Direction**	**References**
Protein Function	Structure determines function	Dynamics enables function	Revolutionary	Very High	Energy-based design	[2], [5], [315]
Catalysis	Transition state stabilization	Energy flow assistance	Transformative	High	Dynamic engineering	[10], [11], [20]
Drug Design	Lock and key binding	Ensemble modulation	Major revision	High	Allosteric focus	[282], [283], [284]
Evolution	Sequence-structure selection	Dynamic property optimization	Conceptual expansion	Medium-High	Predictive frameworks	[221], [222], [256]
Cellular Organization	Static molecular machines	Energy flow networks	Fundamental reframing	Emerging	Systems dynamics	[201], [286], [322]

The recognition of proteins as sophisticated machines that harvest and channel thermal energy to perform chemical work represents a profound shift in biological understanding comparable to the most transformative discoveries in the field's history [2], [5]. Just as understanding DNA's structure revealed the mechanism of heredity, recognizing protein dynamics as central to function opens new vistas for manipulating biological systems. The shift from conceptual foundation to established principles over the past three decades has been driven by technological advances that made the invisible visible, theoretical frameworks that connected structure to energetics, and practical applications that demonstrated utility beyond academic interest [315]. The field now stands at an inflection point where fundamental understanding enables rational manipulation, creating unprecedented opportunities in medicine, biotechnology, and synthetic biology. The implications extend beyond proteins to challenge our understanding of cellular organization, suggesting that life maintains order not through static structures but through coordinated energy flow networks that create functional organization from thermal chaos. This perspective aligns biology with fundamental physics, revealing life as a particularly sophisticated example of non-equilibrium systems that maintain organization through continuous energy flux. The next decade promises to be transformative as dynamic principles permeate drug discovery pipelines, enzyme engineering programs, diagnostic development efforts, and synthetic biology designs. We may see the emergence of entirely new fields, such as dynamic medicine, which diagnoses and treats diseases based on aberrant energy flow; protein dynamics engineering, which creates catalysts surpassing nature's solutions; and bioenergetic computing, which exploits conformational changes for information processing. The success of this paradigm shift will depend on continued technological innovation, sustained funding for basic research, training of interdisciplinary scientists, and patience as applications mature from promise to reality. Yet the convergence of evidence, the explanatory power of the framework, and early practical successes suggest that dynamic energy conversion will become as fundamental to biology as the genetic code or protein folding. We stand at the dawn of a new era in molecular biology where the dynamic dance of atoms reveals life's deepest secrets and provides tools to engineer its future.

The synthesis of dynamic principles with classical structural biology heralds unprecedented advances in protein science with cascading benefits for human health, environmental sustainability, and technological innovation, marking a conceptual revolution comparable to the central dogma's establishment as we transition from static molecular snapshots to recognizing proteins as dynamic energy converters, thereby inaugurating scientific and technological frontiers that promise to fundamentally reshape biological understanding and biotechnological capabilities for generations to come.

### 10.6 Implementation roadmap

Implementation of dynamic energy conversion principles will unfold through sequential developmental phases marked by defined milestones and quantifiable success metrics outlined in Table 49, establishing a coordinated framework that synchronizes research initiatives across disciplines while providing benchmarks for tracking advancement from theoretical refinement through experimental validation to practical deployment in therapeutic and biotechnological applications.

**Table 49:** Development timeline for dynamic energy conversion applications.

**Timeframe**	**Milestone**	**Key Developments**	**Success Metrics**	**Potential Barriers**	**References**
Near-term (2–5 years)	AI integration	Structure-dynamics prediction	> 80 % accuracy	Data limitations	[277], [298]
Medium-term (5–15 years)	Commercial applications	Optimized industrial enzymes	> 50 % market share	Regulatory hurdles	[292], [300]
Long-term (15–30 years)	Revolutionary systems	Artificial energy converters	> 95 % efficiency	Technical feasibility	[294], [310]

### 10.7 Concluding perspective: a new understanding of life's molecular machinery

The reconceptualization of proteins from rigid structural scaffolds to sophisticated energy management systems embodies a paradigmatic transformation that celebrates biological complexity while offering actionable frameworks for functional understanding and engineering, with ramifications documented in Table 50 spanning theoretical foundations through practical applications, ultimately revealing how embracing dynamic principles rather than static reductionism unlocks both deeper mechanistic insights and enhanced capabilities for designing next-generation therapeutics and biotechnologies.

**Table 50:** Paradigm shift impact assessment.

**Impact Category**	**Traditional View**	**Dynamic View**	**Transformation Degree**	**Evidence Quality**	**References**
Mechanistic understanding	Structure-function	Energy-function	Revolutionary	High	[2], [5], [315]
Design principles	Static optimization	Dynamic optimization	Transformative	Medium-High	[291], [294]
Therapeutic targeting	Active site focus	Network targeting	Evolutionary	Medium	[282], [283]
Biotechnology applications	Activity enhancement	Efficiency optimization	Significant	High	[292], [298]

The synthesis of dynamic principles with classical structural biology heralds unprecedented advances in protein science with cascading benefits for human health, environmental sustainability, and technological innovation, marking a conceptual revolution comparable to the central dogma's establishment as we transition from static molecular snapshots to recognizing proteins as dynamic energy converters, thereby inaugurating scientific and technological frontiers that promise to fundamentally reshape biological understanding and biotechnological capabilities for generations to come.

## Author statement

I am grateful to the journal's editorial team and the reviewers for dedicating so much time to reviewing this lengthy manuscript. I have made changes to enhance its utility for researchers in this rapidly growing field of interest in biological drug development.

I am willing to seek professional paid services to edit the manuscript for language issues, if it is considered appropriate by the reviewers and editors.

## CRediT authorship contribution statement

**Sarfaraz K. Niazi:** Writing – review & editing, Writing – original draft, Visualization, Investigation, Conceptualization.

## Authorship

The author conceived, developed, and wrote the entire manuscript.

## Funding

No Funding was received.

## Conflict of interest

The author is an advisor to several regulatory agencies, the US Senate, the United Nations and a developer of biological drugs.

## Declaration of Competing Interest

The author is an advisor to the US FDA, EMA, MHRA, the US Senate, the White House, several heads of sovereign states, and a developer of novel biological drugs.
